# Regulation of the Axillary Osmidrosis-Associated ABCC11 Protein Stability by *N*-Linked Glycosylation: Effect of Glucose Condition

**DOI:** 10.1371/journal.pone.0157172

**Published:** 2016-06-09

**Authors:** Yu Toyoda, Tappei Takada, Hiroshi Miyata, Toshihisa Ishikawa, Hiroshi Suzuki

**Affiliations:** 1 Department of Pharmacy, The University of Tokyo Hospital, Tokyo, Japan; 2 RIKEN Center for Life Science Technology, Yokohama, Japan; University of Pittsburgh, UNITED STATES

## Abstract

ATP-binding cassette C11 (ABCC11) is a plasma membrane protein involved in the transport of a variety of lipophilic anions. ABCC11 wild-type is responsible for the high-secretion phenotypes in human apocrine glands, such as that of wet-type ear wax, and the risk of axillary osmidrosis. We have previously reported that mature ABCC11 is a glycoprotein containing two *N*-linked glycans at Asn838 and Asn844. However, little is known about the role of *N*-linked glycosylation in the regulation of ABCC11 protein. In the current study, we investigated the effects of *N*-linked glycosylation on the protein level and localization of ABCC11 using polarized Madin-Darby canine kidney II cells. When the *N*-linked glycosylation in ABCC11-expressing cells was chemically inhibited by tunicamycin treatment, the maturation of ABCC11 was suppressed and its protein level was significantly decreased. Immunoblotting analyses demonstrated that the protein level of the *N*-linked glycosylation-deficient mutant (N838Q and N844Q: Q838/844) was about half of the ABCC11 wild-type level. Further biochemical studies with the Q838/844 mutant showed that this glycosylation-deficient ABCC11 was degraded faster than wild-type probably due to the enhancement of the MG132-sensitive protein degradation pathway. Moreover, the incubation of ABCC11 wild-type-expressing cells in a low-glucose condition decreased mature, glycosylated ABCC11, compared with the high-glucose condition. On the other hand, the protein level of the Q838/844 mutant was not affected by glucose condition. These results suggest that *N*-linked glycosylation is important for the protein stability of ABCC11, and physiological alteration in glucose may affect the ABCC11 protein level and ABCC11-related phenotypes in humans, such as axillary osmidrosis.

## Introduction

Accumulating evidence suggests that ATP-binding cassette (ABC) transporter proteins play a pivotal role in the maintenance of human health [1–5]. Thereby, many studies in recent years have focused on not only the molecular function of each ABC protein but also the regulatory mechanisms responsible for their protein levels and localization in cells. Human ABCC11 (multidrug resistance-associated protein 8, MRP8) is a member of the ABC protein family [6–9]. Its gene encodes a 1382-amino acid transporter protein that contains two ATP-binding cassettes and twelve transmembrane helices. Based on the predicted topology, both the N-terminus and the C-terminus of the ABCC11 protein are thought to be located on the cytoplasmic side of the membrane [7]. Like other ABC transporter proteins [4], ABCC11 is able to transport a variety of compounds across membranes at the cost of ATP hydrolysis. The substrates of ABCC11 include endogenous substances, such as cyclic nucleotides and several sulfated conjugates, as well as drugs such as 5-fluorouracil (5-FU) and methotrexate [9–13]. Transcript analyses revealed that ABCC11 mRNA is ubiquitously expressed in human tissues [14, 15]. Moreover, some immunohistochemical studies demonstrated that the ABCC11 protein is localized on the apical membrane of polarized cells in human apocrine glands *in vivo* [16] and in a cultured cell line expressing recombinant human ABCC11 *in vitro* [17].

Previously, we and collaborators reported that a single nucleotide polymorphism (SNP) in *ABCC11*, 538G>A (rs17822931: Gly180Arg), is the determinant of human earwax [18], a well-known Mendelian trait and one of the apocrine gland-related phenotypes. The 538GG (wild type; WT homozygote) and GA (heterozygote) genotype give a wet phenotype, whereas the 538AA (SNP-type homozygote) gives a dry (secretion-deficient) phenotype. The fact that the 538A allele gives a recessive phenotype is strongly supported by our previous study demonstrating that this SNP variant is recognized as an endoplasmic-reticulum-associated protein degradation (ERAD) substrate and therefore loses its protein function [16]. Furthermore, *ABCC11* 538G>A is reportedly associated with other apocrine gland-related phenotypes, such as apocrine colostrum secretion from mammary glands [19] and axillary osmidrosis [16, 20–22]. In many cases, the 538G allele corresponds to high-secretory phenotypes and the 538A allele corresponds to low-secretory phenotypes, suggesting the involvement of ABCC11 in the physiological regulation of human apocrine glands [8, 16]. Axillary osmidrosis is a distressing condition characterized by strong body odor and profuse sweating from armpits resulting from excessive apocrine secretion. Thus, the inhibition of ABCC11 is considered to be an effective way to prevent and/or treat axillary osmidrosis. However, to date little is known about the regulatory mechanisms of the ABCC11 protein in apocrine glands, although they should include potential targets for ABCC11 inhibition. Accordingly, the investigation of the molecular basis related to the regulation of ABCC11 would be an important issue.

We have previously revealed that ABCC11 is glycosylated at both Asn838 and Asn844, found in the extracellular loop of the ABCC11 protein [16]. Asparagine (*N*)-linked glycosylation is one of the most important post-translational modifications and participates in many cellular mechanisms that contribute to health and disease of humans, such as protein folding, stability, and intracellular trafficking [23, 24]. In fact, being implemented in one of the protein quality control systems, *N*-glycosylation affects the protein level and/or localization of physiologically important transporters [25–29]. At present, however, the role of *N*-linked glycosylation in the regulation of ABCC11 is not well understood, although the glycosylation is a known post-translational modification of ABCC11. Regarding this unsolved issue, our previous study was restricted in the determination of *N*-linked glycosylation sites in ABCC11 expressed only in non-polarized Flp-In-293 cells [16]. Considering that the physiological expression of ABCC11 protein has been confirmed only in polarized cells in human body, we should investigate the effect of *N*-linked glycan on the protein expression, stability, and localization of ABCC11 using polarized cells.

In the present study, we expressed ABCC11 WT and *N*-linked glycosylation-deficient N838Q and/or N844Q mutant(s) in Madin-Darby canine kidney II (MDCKII) cells, and then compared the protein levels and localization of each ABCC11 mutant. The disruption of *N*-linked glycosylation resulted in the decrease of the ABCC11 protein level and enhanced protein degradation, whereas exclusion of *N*-linked glycan did not affect the localization of ABCC11. These findings suggest the role of *N*-linked glycan in increasing the protein stability of ABCC11 in polarized cells, a cell type that the physiological expression of ABCC11 protein has been confirmed.

## Materials and Methods

### Materials

The following compounds were purchased from commercial sources that are indicated in parentheses: tunicamycin and bafilomycin A_1_ (Wako Pure Chemical Industries Ltd., Tokyo, Japan); 5-fluorouracil (5-FU) and cycloheximide (Nacalai Tesque, Inc., Kyoto, Japan); Peptide *N*-glycosidase F (PNGase F) (New England Biolabs, Inc., Ipswich, MA, USA); MG132 (Calbiochem, Darmstadt, Germany). All other chemicals used were commercially available and of analytical grade.

### Construction of ABCC11-containing expression vectors

The full-length ABCC11 WT (NCBI accession; NM_033151) open reading frame (ORF) in pcDNA3.1/hygro(-) plasmid, constructed in our previous study [16], was amplified with the *Sal* I site attached at the 5′-end and with the *BamH*I site at the 3′-end by PCR, and then inserted into the pEGFP-C1 vector plasmid (Clontech Laboratories, Inc., Palo Alto, CA, USA) for CMV-driven expression of EGFP-ABCC11 in mammalian cells. In a similar manner, ABCC11 ORF without the termination codon was inserted into the *Sal* I and the *BamH*I sites of the pEGFP-N1 vector (Clontech Laboratories, Inc.) for the expression of ABCC11-EGFP. As a negative or positive control protein for localizing on the apical membrane of MDCKII cells, we chose a glucose transporter type 9 isoform a (long form variant) and b (short form variant) (GLUT-9a and b, also known as solute carrier family 2 member 9; SLC2A9a and b, NCBI accession; NM_020041.2 and NM_001001290, respectively) [30] and constructed the expression vector for GLUT-9a-EGFP and GLUT-9b-EGFP with a similar strategy, respectively. As another positive control gene in ABC transporter family, we used ABCG2 (NCBI accession; NM_004827) ORF in pEGFP-C1 vector (Clontech Laboratories, Inc.) for the expression of EGFP-ABCG2 [31]. Using a site-directed mutagenesis technique, several mutants of ABCC11 lacking the *N*-linked glycosylation site(s) (N838Q, N844Q, N838/844Q) were constructed with a pEGFP-N1 vector according to our previous study [16]. The construct of *N*-linked glycosylation-deficient ABCG2 mutant (N596Q) was constructed with a similar strategy. Introduction of mutations was confirmed by full sequencing using BigDye® Terminator v3.1 (Applied Biosystems Inc., Foster City, CA, USA) with Applied Biosystems® 3130 Genetic Analyzer (Applied Biosystems Inc.) according to the manufacturer’s protocol.

### Cell culture and transfection

MDCKII cells were maintained in Dulbecco’s Modified Eagle’s Medium (DMEM, 4.5 g/L glucose with L-Gln and sodium pyruvate) (Nacalai Tesque) supplemented with 10% fetal bovine serum (FBS) (Biowest, Nuaillé, France), and 1% penicillin-streptomycin (Nacalai Tesque) at 37°C in a humidified atmosphere of 5% (v/v) CO_2_ in air as described previously [32]. Each vector plasmid for ABCC11 WT or its mutants was transfected into MDCKII cells by using polyethyleneimine MAX (PEI-MAX) (Polysciences Inc., Warrington, PA, USA) as described previously [33] with some modification. The amount of plasmid DNA used for transfection was adjusted to be the same for ABCC11 WT and its mutants. In brief, each plasmid was mixed with PEI-MAX (1 μg of plasmid/5 μL of PEI-MAX for 5 × 10^5^ cells of MDCKII) in Opti-MEM^TM^ (Invitrogen, Carlsbad, CA, USA) and incubated for 20 min at room temperature. MDCKII cells were collected after the treatment with a 2.5 g/L-Trypsin and 1 mmol/L-EDTA solution (Nacalai Tesque), followed by centrifugation at 1,000 × *g* for 5 min. The cell pellet was re-suspended in fresh DMEM, and the resulting suspension was mixed with plasmid/PEI-MAX mixture (50:50, v/v). Then, the MDCKII cells were re-seeded onto cell culture plates at a concentration of 1.4 × 10^5^ cells/cm^2^. The medium was replaced with fresh medium after the first 8 h of incubation. For the selection and maintenance of stable transfectants (MDCKII/ABCC11 WT-EGFP), transfected cells were cultured in the presence of 2.5 mg/mL G418 sulfate (Nacalai Tesque). To examine the effect of glucose level in culture medium on the ABCC11, DMEM with glucose (1.0 g/L (low) and 4.5 g/L (high)) with L-Gln and sodium pyruvate, (Nacalai Tesque) supplemented with 10% FBS and 1% penicillin-streptomycin was used in the experiments.

Human embryonic kidney 293 (HEK293) cells (Life technologies, Tokyo, Japan) and 293A cells (Invitrogen) were maintained in DMEM supplemented with 10% FBS, 1% penicillin-streptomycin, 2 mM L-Glutamine (Nacalai Tesque), and 1 × Non-Essential Amino Acid (Life Technologies) in a similar manner as described above. Before transfection, HEK293 cells were seeded onto cell culture plates at a concentration of 1.4 × 10^5^ cells/cm^2^. Twenty-four hours after the seeding, each plasmid vector was transiently transfected to the cells using PEI-MAX (1 μg of plasmid/5 μL of PEI-MAX in Opti-MEM^TM^). The medium was replaced with fresh medium after the first 8 h of incubation.

### Construction and infection of recombinant adenovirus

Recombinant adenovirus for the expression of human ABCC11 WT was constructed using a ViraPower^TM^ Adenovial Gateway^TM^ Expression Kit (Invitrogen) according to the manufacturer’s protocol. In brief, the full-length ABCC11 WT ORF was inserted into pAd/CMV/V5-DEST^TM^ Gateway Vector plasmid. After the digestion of the plasmid with *Pac* I (New England Biolabs) treatment for two hours at 37°C, the linearized construct containing the plasmid region for the proper packaging and production of adenovirus and the expression of non-tagged ABCC11 was purified, and then used to transfect into 293A adenovirus producer cells. Several days after the transfection, a crude viral lysate was prepared from the harvest cells and used for the infection of newly seeded 293A cells to amplify the adenovirus. After the amplification, the adenovirus was purified using an Adenovirus (Ad5) Purification and Concentration Kit (AdenoPACK 20; Sartorius, Goettingen, Germany) according to the manufacturer’s instruction, and stored at -80°C until use. Then, the resulting adenovirus titer was determined using an Adeno-X™ Rapid Titer Kit (Clontech). As a control adenovirus, EGFP-expressing adenovirus was constructed with a similar strategy.

MDCKII cells were plated onto cell culture plates at a density of 1.4 × 10^5^ cells/cm^2^. After 12 h, cells were infected with recombinant adenoviruses harboring non-tagged human ABCC11 WT or EGFP at indicated multiplicity of infections (MOIs) as described previously [34]. At 48 h after the infection, the culture medium was replaced with fresh medium with or without tunicamycin, and the cells were cultured for further 24 h.

### Preparation of whole cell lysate

At indicated times after the plasmid transfection, MDCKII cells or HEK293 cells were washed by ice-cold phosphate-buffered saline without potassium; PBS (-) twice, and were harvested using a cell scraper. After centrifugation at 800 × *g* for 5 min, cells were treated with cell lysis buffer A containing 50 mM Tris/HCl (pH 7.4), 1 mM dithiothreitol, 1% (v/v) Triton X-100, and a protease inhibitor cocktail for general use (Nacalai Tesque). The cell suspension samples were homogenized by passage through a 27-gauge needle equipped with 1 mL disposable plastic syringe (Terumo Corp., Tokyo, Japan) 10 times. The homogenate was centrifuged at 3,000 × *g* at 4°C for 10 min and the resulting supernatant (whole cell lysate) was collected in a new tube. Protein concentration of whole cell lysate was quantified using BCA Protein Assay Kit (Pierce, Rockford, IL, USA) with BSA as a standard according to the manufacturer’s protocol.

### Glycosidase treatment

For glycosidase treatment, the whole cell lysate samples were incubated with PNGase F (1.25 U/μg of protein) at 37°C for 10 min as described previously [16], and then subjected to immunoblotting analysis. In the current study, the levels of total ABCC11 protein (glycosylated and non-glycosylated) in cells were determined using PNGase F-treated samples. The PNGase F-treated sample was used also as a control indicating the band position for non-glycosylated ABCC11 in immunoblotting analyses.

### Immunoblotting analysis

EGFP-fused ABCC11 WT and its mutants expressed in MDCKII cells were detected by immunoblotting with a rabbit anti-EGFP polyclonal antibody (A11122; Life technologies). Non-tagged ABCC11 expressed in HEK293 cells and ABCC11-expressing adenovirus-infected MDCKII cells was detected with a rat anti-ABCC11 monoclonal antibody (M8I-74; Abcam Inc., Cambridge, MA, USA). In brief, at first, whole cell lysate samples with or without PNGase F treatment were mixed with SDS-PAGE sample buffer solution containing 10% (v/v) 2-mercaptoethanol. Thereafter, samples were electrophoretically separated on 7.5% (v/v) poly-acrylamide gels, and then transferred to Hybond® ECL^TM^ (enhanced chemiluminescence) nitrocellulose membrane (GE Healthcare UK Ltd., Buckinghamshire, UK) by electroblotting at 15 V for 70 min. For blocking, the membrane was incubated with Tris-buffered saline containing 0.05% (v/v) Tween 20 (TTBS) and 5% (w/v) skim milk, at 4°C overnight.

Immunoblotting was performed by using the anti-GFP antibody diluted 1,000-fold in TTBS containing 5% skim milk as the first antibody and a donkey anti-rabbit IgG-horseradish peroxidase (HRP)-conjugate (NA934V; GE Healthcare UK Ltd.) diluted 3,000-fold as the second antibody. To detect α-tubulin protein as an internal loading control, we used a rabbit anti-α-tubulin antibody (ab15246; Abcam Inc.) diluted 1,000-fold as the first antibody and the donkey anti-rabbit IgG-HRP-conjugate diluted 3,000-fold as the second antibody. To detect non-tagged ABCC11 protein, we used the anti-ABCC11 antibody diluted 100-fold as the first antibody and a goat anti-rat IgG-HRP-conjugated (NA935V; GE Healthcare UK Ltd.) diluted 2,000-fold as the second antibody. For the detection of 135-kD glycoprotein (gp135) expressed in MDCKII cells, we used a mouse anti-gp135 monoclonal antibody [35] (3F2/D8; the Developmental Studies Hybridoma Bank (DSHB), Iowa City, IA, USA) diluted 100-fold as the first antibody and a sheep anti-mouse IgG-HRP-conjugated (NA931V; GE Healthcare UK Ltd.) diluted 3,000-fold as the second antibody. The 3F2/D8 was deposited to the DSHB by Ojakian, G.K. (DSHB Hybridoma Product 3F2/D8). HRP-dependent luminescence was developed with ECL^TM^ Prime Western Blotting Detection Reagent (GE Healthcare UK Ltd.) and detected using a multi-imaging Analyzer Fusion Solo 4^TM^ system (Vilber Lourmat, Eberhardzell, Germany). The band density was quantified with Fusion software (Vilber Lourmat) to assess the protein expression levels of ABCC11 WT and its mutants.

### Cell staining and confocal laser scanning microscopic observation

For confocal laser scanning microscopic observation, MDCKII cells transiently transfected with various ABCC11-containing vectors or control vector were plated onto a collagen-coated glass bottom dish (cover size 22 × 22 mm and 0.16–0.19 mm thick; Matsunami Glass Inc., Tokyo, Japan), 72 h prior to the experiments. After fixation with ice-cold methanol for 10 min, and washed three times with PBS (-). For the staining of endogenous gp135 protein in MDCKII cells as an apical membrane marker [36], fixed-MDCKII cells were incubated with the mouse anti-gp135 monoclonal antibody diluted 100-fold in PBS (-) containing 1% BSA (BSA-PBS) as the first antibody for one hour at room temperature, then washed three times with BSA-PBS. Subsequently, cells were further incubated with a goat anti-mouse IgG-Alexa Fluor^®^ 546-conjugate (A11003; Invitrogen) as the second antibody for one hour at room temperature in the dark. After the washing by BSA-PBS three times, cells were incubated with TO-PRO®-3 Iodide (Molecular Probes, Eugene, OR, USA) diluted 250-fold in PBS (-) for 10 min at room temperature to visualize the nuclei of cells. Then, the cells were mounted in VECTASHIELD® Mounting Medium (Vector Laboratories, Burlingame, CA, USA). To analyze the localization of EGFP-fused ABCC11 protein, fluorescence was detected using FluoView^TM^ FV1000 confocal laser scanning microscope (Olympus, Tokyo, Japan).

### WST-8 assay

To evaluate cellular resistance to 5-FU, the viability of MDCKII cells stably expressing ABCC11-EGFP or EGFP (mock) were detected using a Cell Count Reagent SF (Nacalai Tesque) according to a manufacturer’s protocol with some modifications as described previously [37]. In brief, cells were seeded at 1 × 10^4^ cells per well into 96-well cell culture plates and pre-cultured for 24 h. After 84 h of incubation with 5-FU at various concentrations, the culture medium was replaced with 100 μL of fresh medium containing 10% (v/v) of the reagent for WST-8 assay. After 2 h of further incubation, the absorbance at 450 nm derived from WST-8-formazan in culture medium was measured using a Varioskan^TM^ Flash Multimode Reader (Thermo Fisher Scientific K.K., Yokohama, Japan).

### Quantification of mRNA expression by real-time PCR

Total RNA was extracted using RNA isoPlus® Reagent (Takara, Shiga, Japan) from MDCKII cells, according to the manufacturer’s protocol. First-strand cDNA was prepared from the extracted total RNA in a reverse transcriptase reaction with ReverTra Ace® qPCR RT Kit (TOYOBO, Osaka, Japan), and was used as a template for the following quantitative RT-PCR (qRT-PCR). To examine the mRNA levels of ABCC11 and β-actin, the cDNA was amplified by qRT-PCR with SYBR® GreenER^TM^ qPCR SuperMix Universal (Life Technologies) in an Eco Real-Time PCR System (Illumina, San Diego, CA, USA) using the following specific primer sets: human ABCC11 (5′-GACAGAGATTGGAGAGCGGG-3′; 5′-CTGACGGTCGGAATAGACGG-3′); β-actin (5′-CCGGAAGGAAAACTGACAGC-3′; 5′-GTGGTGGTGAAGCTGTAGC-3′). The qRT-PCR reaction was initiated with incubation at 50°C for 2 min, followed by 95°C for 10 min. The cycling protocol consisted of 2 steps: at 95°C for 15 sec and 60°C for 1 min, for 40 cycles. Finally, an additional incubation was performed for the construction of a dissociation curve. Acquisition of qRT-PCR data and subsequent analyses were carried out using Eco software ver 3.0 (Illumina). The expression levels of each gene were calculated using a standard curve prepared by serial dilution of the reference sample. The expression levels of ABCC11 were normalized by those of β-actin.

### ABCC11 protein stability

Cycloheximide, an inhibitor of protein translation, was added at 72 h after plasmid transfection to MDCKII cells, and then the cells were collected at 0, 3, 6, and 12 h. MG132, an inhibitor of proteasomal protein degradation, or bafilomycin A_1_, an inhibitor of lysosomal protein degradation, was added at 48 h after the transfection, and the cells were cultured for further 24 h. The protein levels of ABCC11 in each sample were examined by immunoblotting analysis as described above.

### Multiple sequence alignment

Amino acid sequences of ABCC11 in several species were obtained from NCBI Entrez database. Multiple sequence alignment and homology calculation were carried out using the GENETYX ver.9 software (GENETYX Co., Tokyo, Japan) with the ClustalW 2.1 Windows program according to the manufacturer’s instruction.

### Statistical analysis

All statistical analyses were performed by using EXCEL 2013 (Microsoft Corp., Redmond, WA, USA) with Statcel3 add-in software (OMS publishing Inc., Saitama, Japan). Different statistical tests were used for different experiments as described in figure legends. The significance of each value was determined when the *P* value was less than 0.05 and 0.01.

## Results

### Expression of EGFP-fused ABCC11 in MDCKII cells

**Fig 1:**
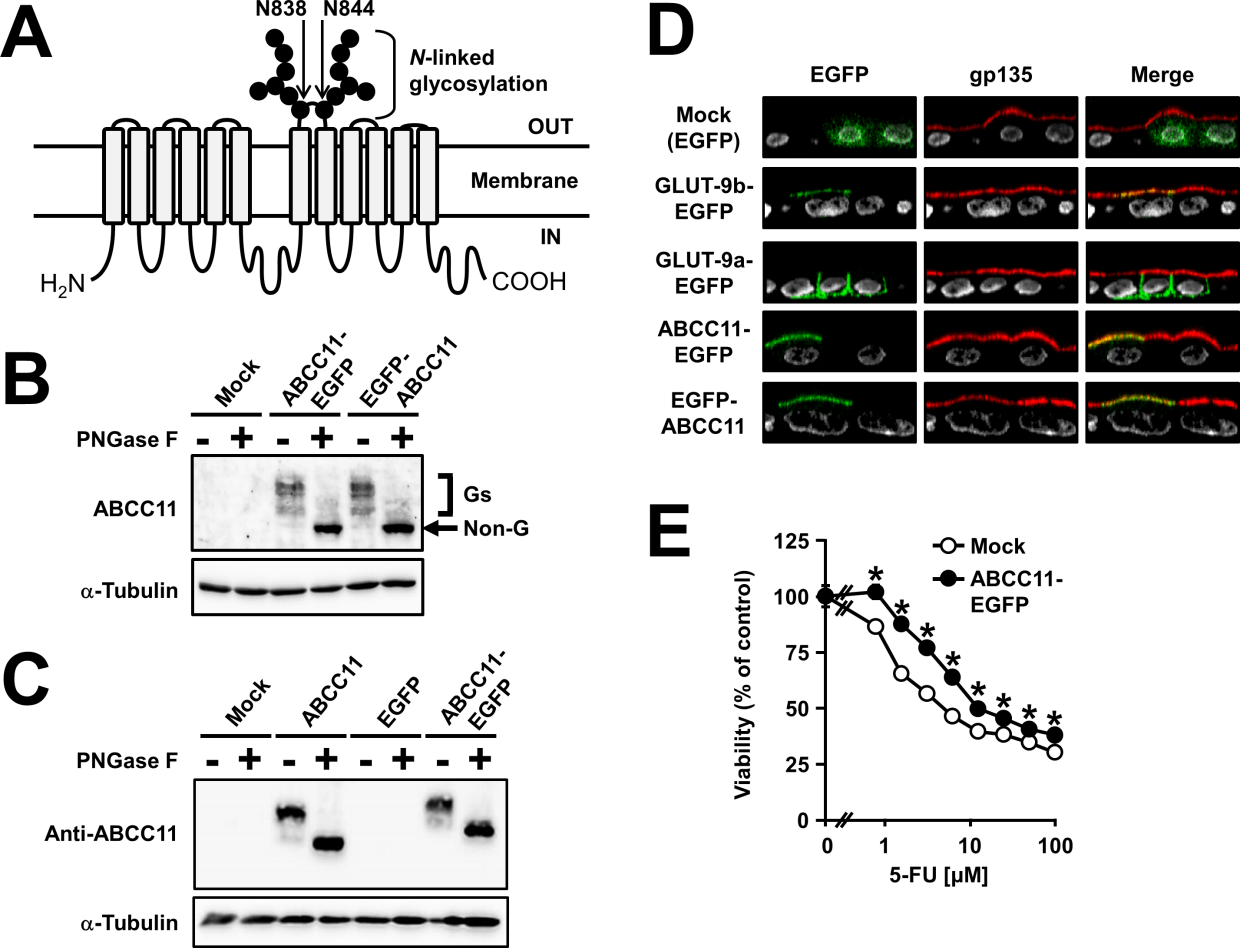
Characterization of ABCC11 fused with EGFP, expressed in MDCKII cells. (A) Schematic illustration of ABCC11 protein. ABCC11 has two *N*-linked glycosylation sites, N838 and N844, in an extracellular loop between transmembrane helices 7 and 8. (B) Glycosylation status and expression of ABCC11-EGFP and EGFP-ABCC11 in MDCKII cells. Cell lysates were prepared 72 h after the transfection, and subsequently subjected to immunoblotting after treatment with or without PNGase F. The immunoreactive bands, corresponding to the glycosylated forms (Gs) of EGFP-fused ABCC11 protein, were disappeared after PNGase F treatment. The apparent molecular weight value of the non-glycosylated form (Non-G) of EGFP-fused ABCC11 protein was about 180,000. α-Tubulin: a loading control. (C) Glycosylation status and expression of non-tagged ABCC11 and ABCC11-EGFP in HEK293 cells. Cell lysates were prepared 72 h after the transfection and treated with or without PNGase F, and subsequently subjected to immunoblotting using the anti-ABCC11 antibody. α-Tubulin: a loading control. (D) Apical localization of ABCC11-EGFP and EGFP-ABCC11 in MDCKII cells 72 h after the transfection. An endogenous apical membrane marker gp135 was immunostained using anti-gp135 antibody (red). Nuclei were stained with TO-PRO®-3 iodide (gray). All panels show the Z-sectioning images. Mock (EGFP): a control vector; GLUT-9b-EGFP: a positive control for apical localization; GLUT-9a-EGFP: a negative control for apical localization. (E) 5-fluorouracil (5-FU) resistance activity of MDCKII/mock cells and MDCKII/ABCC11 WT-EGFP cells. Data are expressed as mean ± S.E.M. *n* = 8. Where *vertical bars* are not shown, the S.E.M. was contained within the limits of the symbol. Statistical analyses for significant differences were performed according to Student’s *t* test (*, *P* < 0.01 compared with mock).

In order to visualize human ABCC11 protein in MDCKII cells, we constructed two types of expression vectors for ABCC11 fused with EGFP at the C-terminal (ABCC11-EGFP) or N-terminal (EGFP-ABCC11) in the cytosolic domain of ABCC11 (**Fig 1A**). We first characterized each EGFP-fused ABCC11 protein expressed in MDCKII cells. Expression of human ABCC11 WT fused with EGFP was examined by immunoblotting analysis (**Fig 1B**) 72 h after the transfection. As shown in **Fig 1B**, both ABCC11-EGFP and EGFP-ABCC11 were detected as two bands, and PNGase F treatment shifted these immunoreactive bands to another, lower band with a molecular weight of about 180,000. This molecular weight is consistent with that estimated from each cDNA of ABCC11 fused with EGFP, suggesting that the position of the EGFP-tag did not affect the pattern of ABCC11 glycosylation in MDCKII cells. Unfortunately, non-tagged ABCC11 induced by plasmid transfection into MDCKII cells was undetectable with immunoblotting using the anti-ABCC11 antibody (**S1 Fig**). This result was probably due to the lower sensitivity of the anti-ABCC11 antibody as compared with the anti-EGFP antibody and the low efficiency of plasmid transfection in MDCKII cells. However, both non-tagged ABCC11 protein and ABCC11-EGFP protein transiently expressed in HEK293 cells were detected by immunoblotting using the anti-ABCC11 antibody (**Fig 1C**) as similar intensity and band pattern except for the different estimated molecular weight, suggesting that the presence of EGFP-tag did not affect the glycosylation pattern and protein level of glycosylated-ABCC11 protein. In order to examine the localization of ABCC11 in MDCKII cells, we performed confocal microscopic observation of MDCKII cells 72 h after the transfection (**Fig 1D**). At that time, EGFP-tagged GLUT-9b, an apical membrane protein in kidney cells [30], was co-localized with endogenous gp135 protein, an apical membrane marker in MDCKII cells, whereas EGFP-tagged GLUT-9a, a basal membrane protein in kidney cells [30], was not co-localized with gp135. These results demonstrate the successful construction of polarization and the presence of apical sorting system in MDCKII cells used in the present study. Furthermore, both ABCC11-EGFP and EGFP-ABCC11 showed an apical localization in MDCKII cells (**Fig 1D**), as did GLUT-9b-EGFP. This result reflects the physiological localization of ABCC11 in polarized cells in apocrine glands [16] and is consistent with the previous report [17]. In addition, MDCKII cells stably expressing ABCC11-EGFP were more resistant to 5-FU, an anti-cancer drug, than mock MDCKII cells which were constructed with a pEGFP-N1 vector (**Fig 1E**). Considering the ability of ABCC11 to efflux 5-FU and its active metabolite 5-fluoro-2′-deoxyuridine 5′-monophosphate [10], this drug resistance suggests that ABCC11-EGFP is expressed in MDCKII cells in its functional form. Therefore, we used the construct of ABCC11-EGFP in the following studies.

### Complete absence of *N*-linked glycosylation decreases ABCC11 protein levels

**Fig 2:**
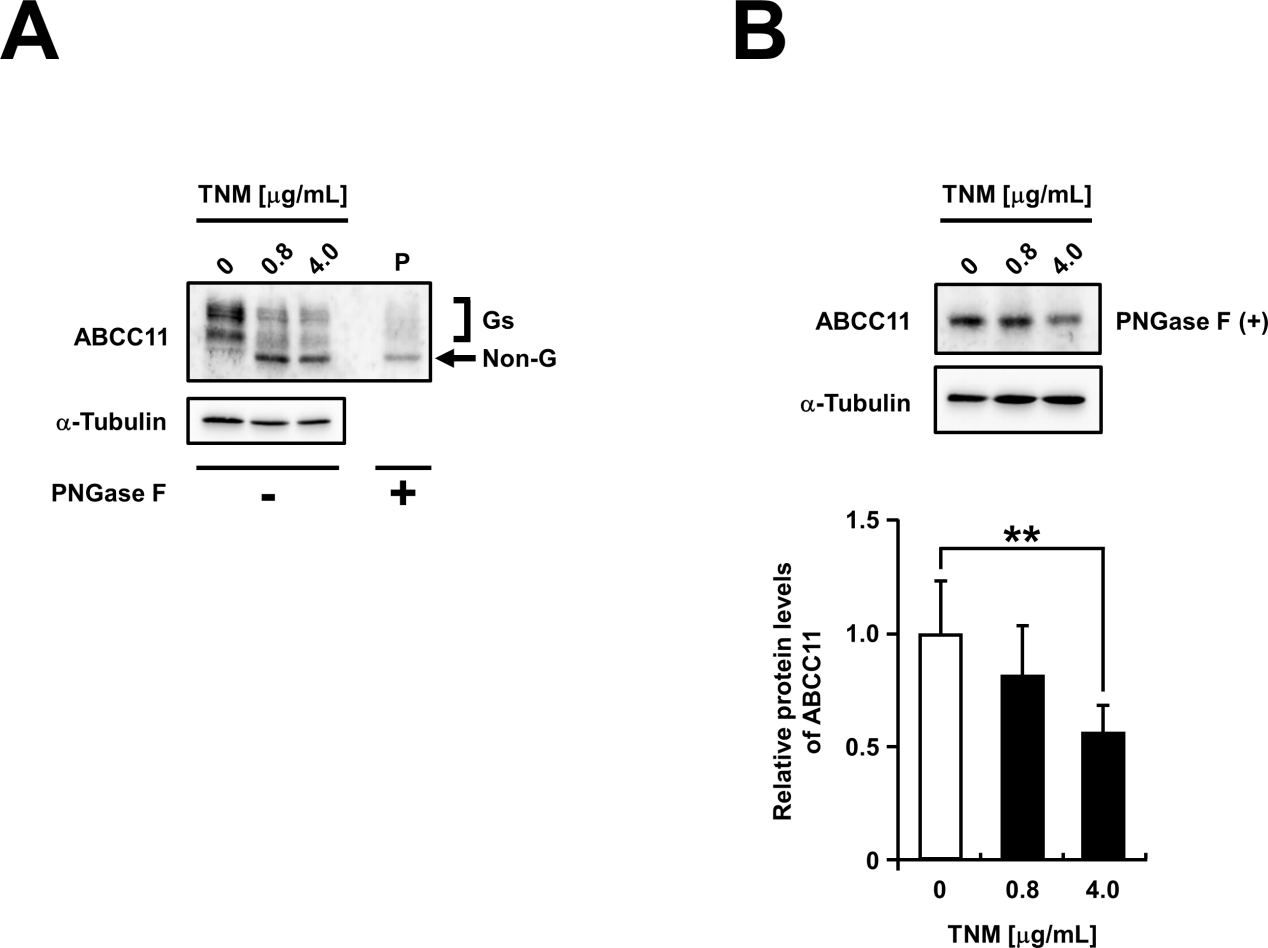
The effects of tunicamycin, an *N*-linked glycosylation inhibitor, on lowering the protein levels of ABCC11. Forty-eight hours after the transfection, MDCKII cells transiently expressing ABCC11 wild-type (WT) were cultured with tunicamycin (TNM) (0, 0.8, and 4.0 μg/mL) for further 24 h, then cell lysate samples were prepared after the treatment without (A) or with (B) PNGase F, and then subjected to immunoblotting. α-Tubulin: a loading control. P: PNGase F-treated ABCC11 WT as a control for the band position of non-glycosylated ABCC11 (details are described in *Materials and Methods*). The signal intensity ratio (ABCC11/α-tubulin) of the immunoreactive bands was determined and normalized to that in TNM-untreated cells (0 μg/mL in B). Data are expressed as mean ± S.D. *n* = 5. Statistical analyses for significant differences were performed according to Student’s *t* test (**, *P* < 0.01 vs control).

In order to investigate the significance of *N*-linked glycosylation in the regulation of ABCC11 protein, we conducted biochemical evaluations. At first, we examined the effect of tunicamycin, an *N*-linked glycosylation inhibitor, on the protein levels of ABCC11. The tunicamycin-treatment of MDCKII cells transiently expressing ABCC11-EGFP blocked ABCC11 glycosylation, and shifted the higher molecular weight bands (glycosylated forms) to a lower band with a molecular weight of about 180,000 (non-glycosylated form) (**Fig 2A**). To analyze the effect of tunicamycin on the protein levels of ABCC11, we treated all samples with PNGase F to remove *N*-linked glycan, resulting in the detection of a single band in immunoblotting (**Fig 2B**). Quantitative evaluation of the single bands revealed that the protein levels of ABCC11 in the tunicamycin-treated cells were lower than those in non-treated cells (**Fig 2B**). Furthermore, we examined the effect of tunicamycin treatment on the protein levels of non-tagged ABCC11 in ABCC11-expressing adenovirus-infected MDCKII cells (**S2 Fig**). The result shows that the protein levels of non-tagged ABCC11 in the tunicamycin-treated cells were significantly lower than those in non-treated cells.

Since the transfection efficiency in MDCKII cells was low (**S1 Fig**), we confirmed that our experimental condition was enough to evaluate the effect of *N*-linked glycosylation on the total protein levels of another ABC transporter ABCG2: WT and Q596, an *N*-linked glycosylation deficient mutant (**S3 Fig**). As expected, ABCG2 protein levels of the Q596 mutant were significantly lower than those of the WT, and each ABCG2 protein was mainly localized on the apical membrane of MDCKII cells. These results were consistent with the previous reports showing the contribution of *N*-linked glycosylation to the protein stability of ABCG2 without any apparent effect on the apical localization of ABCG2 [27, 31].

**Fig 3:**
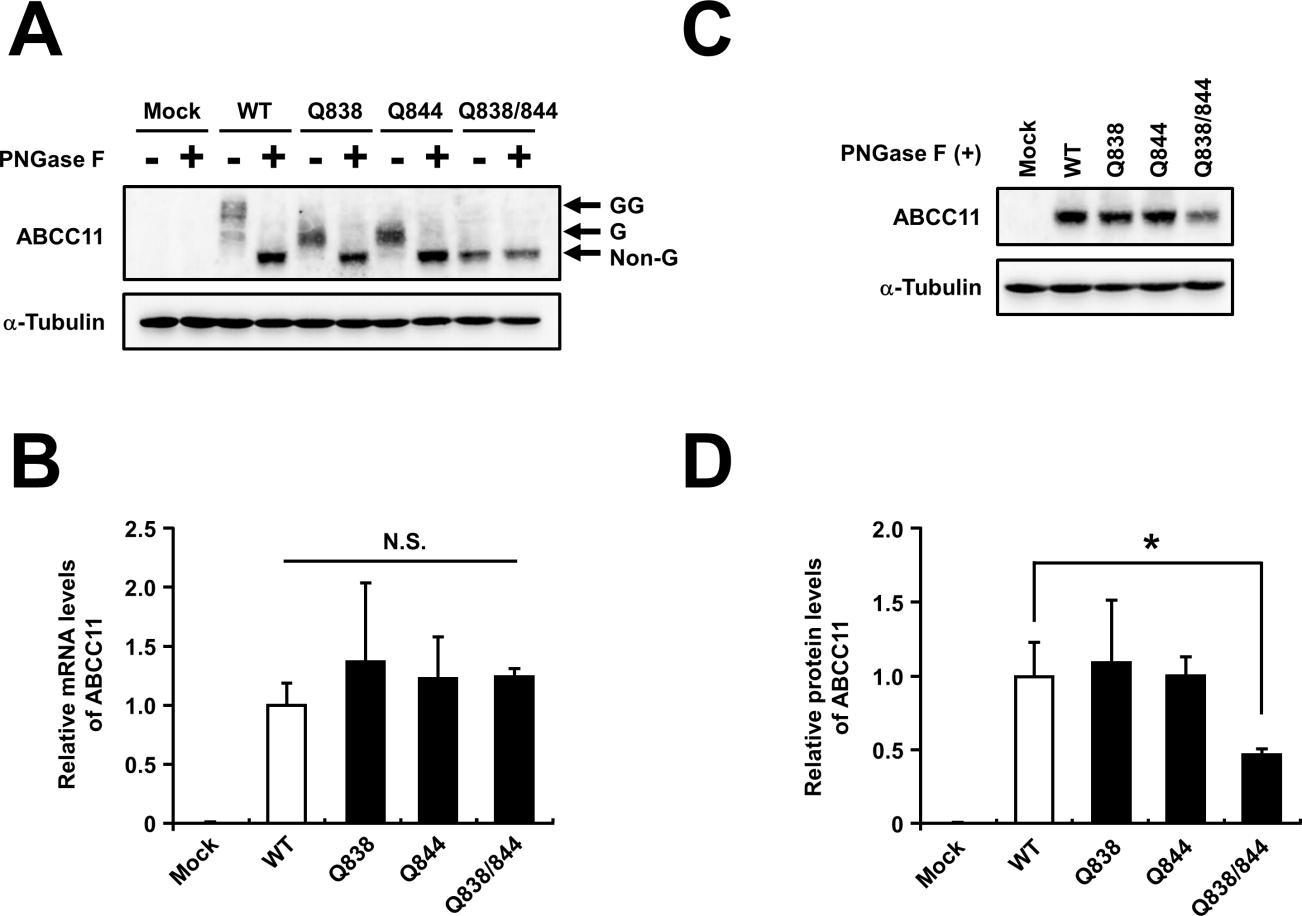
The effect of the disruption of *N*-linked glycosylation sites on the protein levels of ABCC11. (A) Glycosylation status and expression of ABCC11 wild-type (WT) and its mutants expressed in MDCKII cells 72 h after the transfection. The immunoreactive bands, corresponding to the glycosylated forms (G, glycosylation at N838 or N844; GG, at both N838 and N844) of the ABCC11 protein, disappeared with PNGase F treatment. α-Tubulin: a loading control. (B) mRNA levels of ABCC11 WT and its mutants. The mRNA levels were normalized to the WT (control) level. (C and D) Densitometoric analysis of protein levels of ABCC11 WT and glycosylation-deficient mutants. After PNGase F treatment, cell lysate samples were subjected to immunoblotting. The signal intensity ratio (ABCC11/α-tubulin) of the immunoreactive bands (C) was determined and normalized to the WT level. Data are expressed as mean ± S.D. *n* = 4. Statistical analyses for significant differences were performed according to Dunnett’s test (*, *P* < 0.05; N.S.: not significantly different as compared with WT).

To further examine the potential role of *N*-linked glycan in the stability of ABCC11 protein, we constructed three mutants of ABCC11 lacking *N*-glycosylation site(s). Since ABCC11 protein has two glycosylation sites, N838 and N844 (**Fig 1A**), either one or both asparagine (N) residues were substituted by glutamine (Q). Immunoblotting analysis demonstrated that, without PNGase F treatment, the molecular weights of all mutants of ABCC11 (Q838, Q844, Q838/844) were lower than that of ABCC11 WT (**Fig 3A**). In ABCC11 WT, Q838, and Q844, higher molecular weight bands were disappeared and a lower band was appeared with PNGase F treatment (**Fig 3A**). On the other hand, the band pattern of the Q838/844 mutant was not changed by PNGase F treatment (**Fig 3A**), indicating that this mutant completely loses the addition of *N*-linked glycans. At that time, mRNA levels of each ABCC11 were not significantly different (**Fig 3B**). Immunoblotting analysis addressing PNGase F-treated samples revealed that the protein level of ABCC11 Q838/844 was lower than that of ABCC11 WT (**Fig 3C**), whereas the protein levels of ABCC11 Q838 and Q844 mutants which each retain one *N*-glycosylation site showed no difference from that of ABCC11 WT. Densitometric analysis revealed that the ABCC11 protein level of the Q838/844 mutant was about half that of the WT (**Fig 3D**). This result is consistent with the tunicamycin-dependent decrease of ABCC11 protein level (**Fig 2B**). Accordingly, at least one *N*-glycan is necessary for the efficient expression of ABCC11.

### *N*-linked glycosylation is not essential for ABCC11 trafficking to apical membrane

**Fig 4:**
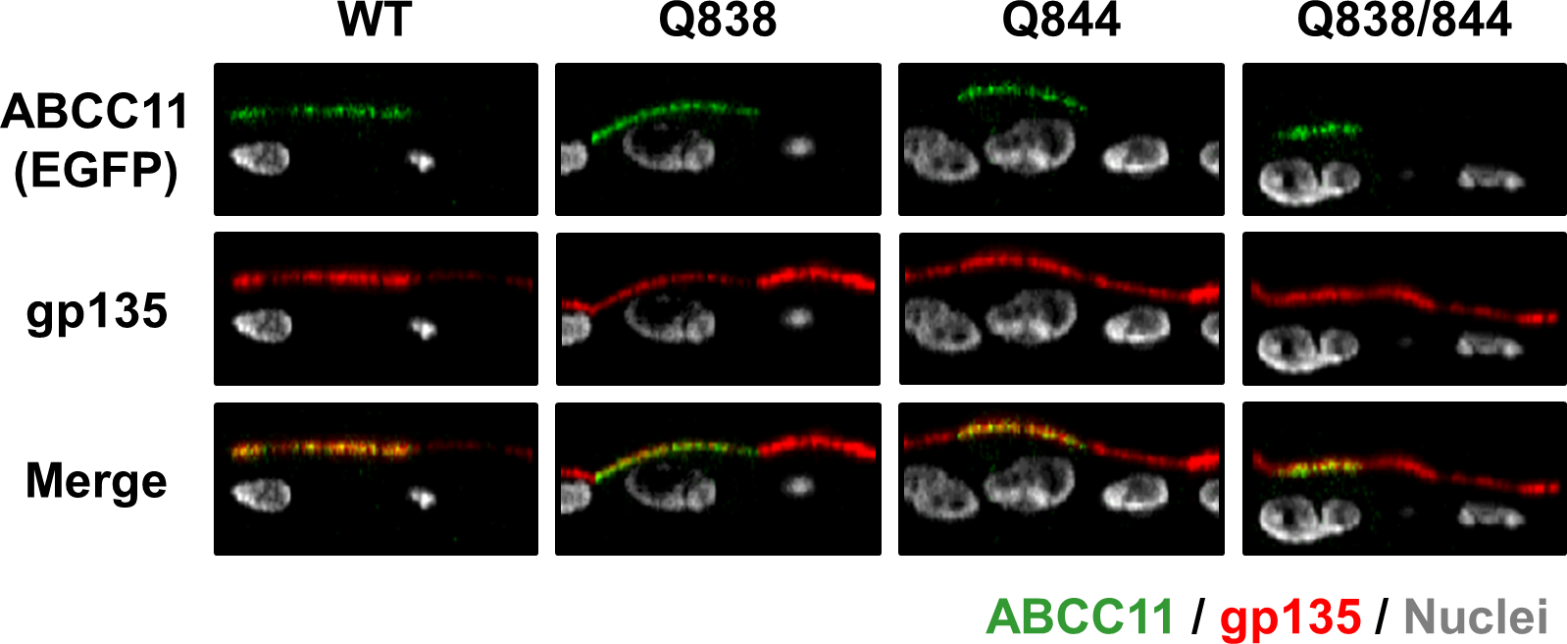
The effect of the disruption of *N*-linked glycosylation sites on the localization of ABCC11. Confocal microscopic images were obtained 72 h after the transfection. All samples show the apical localization of ABCC11 in MDCKII cells. An endogenous apical membrane marker gp135 was immunostained using the anti-gp135 antibody (red). Nuclei were stained with TO-PRO®-3 iodide (gray). All panels show the Z-sectioning images.

*N*-linked glycans attached to the extracellular domain of membrane proteins are often important for protein apical localization [38, 39]. Therefore, we examined the cellular localization of each ABCC11 mutant lacking one or two (all) *N*-glycans. Confocal microscopic observations revealed that all mutants (Q838, Q844, and Q838/844) localized on the apical membrane of MDCKII cells as ABCC11 WT did (**Fig 4**). These results suggest that the absence of *N*-linked glycan did not affect the cellular localization of ABCC11 protein in MDCKII cells. Hence, *N*-linked glycosylation could not be essential for ABCC11 trafficking to apical membrane but important for the stability of ABCC11 protein, at least, in MDCKII cells.

### *N*-linked glycosylation of ABCC11 increases protein stability

**Fig 5:**
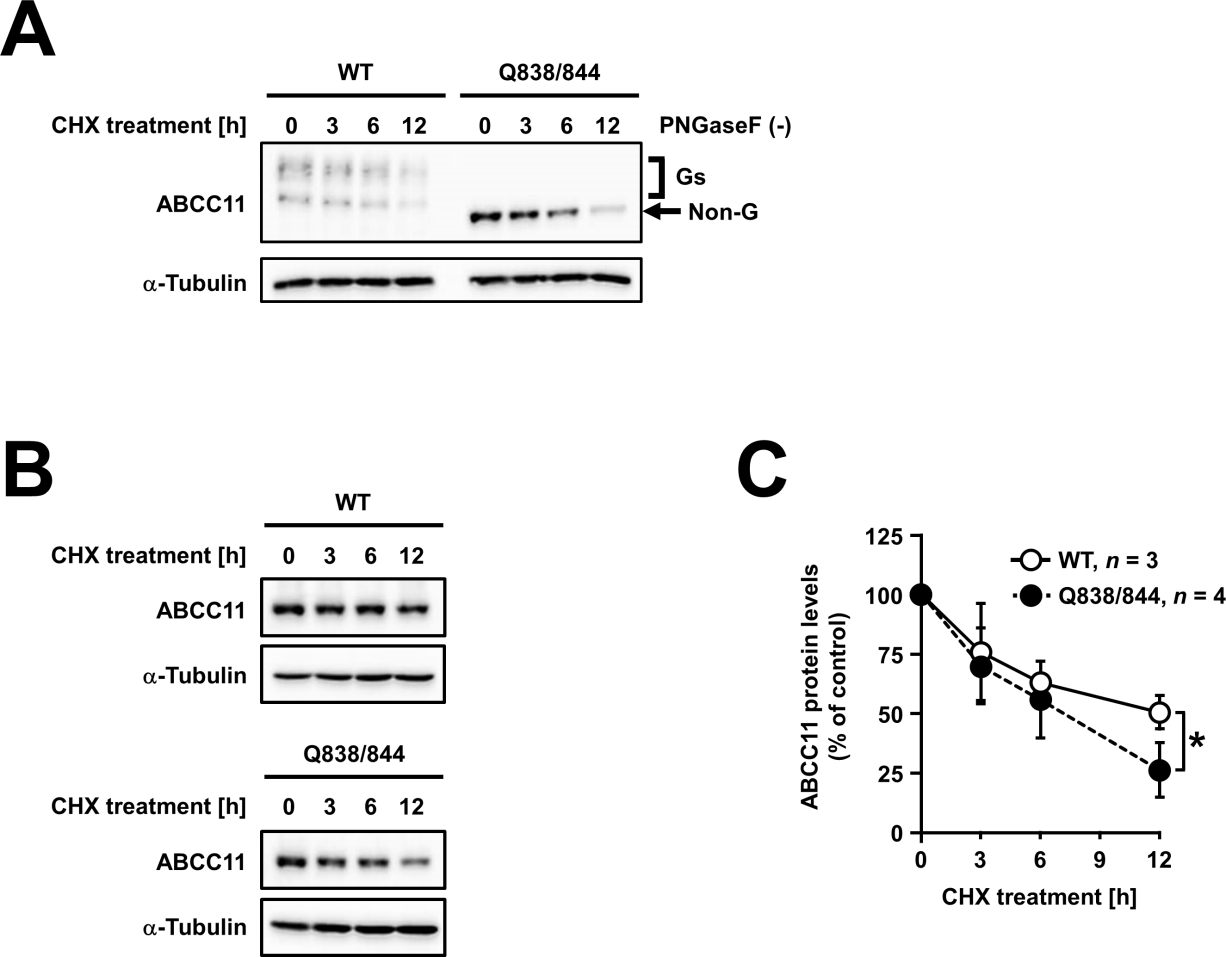
The effect of *N*-linked glycosylation on the protein stability of ABCC11. (A and B) The effect of the inhibition of protein translation with cycloheximide (CHX) on the protein levels of ABCC11 wild-type (WT) and the Q838/844 mutant. Seventy-two hours after the transfection, MDCKII cells transiently expressing ABCC11 WT or Q838/844 were further incubated with 50 μM CHX for 0, 3, 6, and 12 h. At the indicated time, cells were collected and stored at -80°C until use. Simultaneously, all cell lysates were prepared and treated without (A) or with (B) PNGase F, and then subjected to immunoblotting. α-Tubulin: a loading control. The signal intensity ratio (ABCC11/α-tubulin) of the immunoreactive bands corresponding to the non-glycosylated form (Non-G) of ABCC11 protein was determined and normalized to the control (*t* = 0 h) level. Gs, glycosylated forms. (C) Densitometoric analysis of protein levels of the ABCC11 WT and the Q838/844 mutant. Values are expressed as % of control (values at 0 h). Data are expressed as mean ± S.D. *n* = 3 (WT), 4 (Q838/844 mutant). Statistical analyses for significant differences were performed according to Student’s *t* test (*, *P* < 0.05 in indicated time points).

Since the protein level of the *N*-glycosylated ABCC11 was higher than that of non-glycosylated ABCC11 (**Figs 2 and 3**), we next examined whether *N*-linked glycan affects the protein stability of ABCC11. To inhibit *de novo* protein synthesis, we treated MDCKII cells transiently transfected with ABCC11 WT or Q838/844 mutant fused with EGFP with cycloheximide, a protein translation inhibitor, for different periods of time (0, 3, 6, and 12 h). Immunoblotting analysis revealed that the protein level of ABCC11 Q838/844 was decreased faster than that of the ABCC11 WT after treatment with cycloheximide (**Fig 5**). Twelve hours after the cycloheximide treatment, the relative protein level of ABCC11 WT reached about a half of its control (50 ± 7% as compared with *t* = 0 h, estimated half-life was about 12 h) (**Fig 5C**). At the same time point, the relative protein level of the Q838/844 mutant was further decreased (26 ± 12% as compared with *t* = 0 h, estimated half-life was about 6.2 h), indicating that the disruption of *N*-linked glycan enhances the degradation of the ABCC11 protein. Therefore, *N*-linked glycosylation would increase the stability of ABCC11 protein via the protection from protein degradation systems.

### The effect of bafilomycin A_1_ and MG132 on the protein levels of ABCC11 WT and the Q838/844 mutant

**Fig 6:**
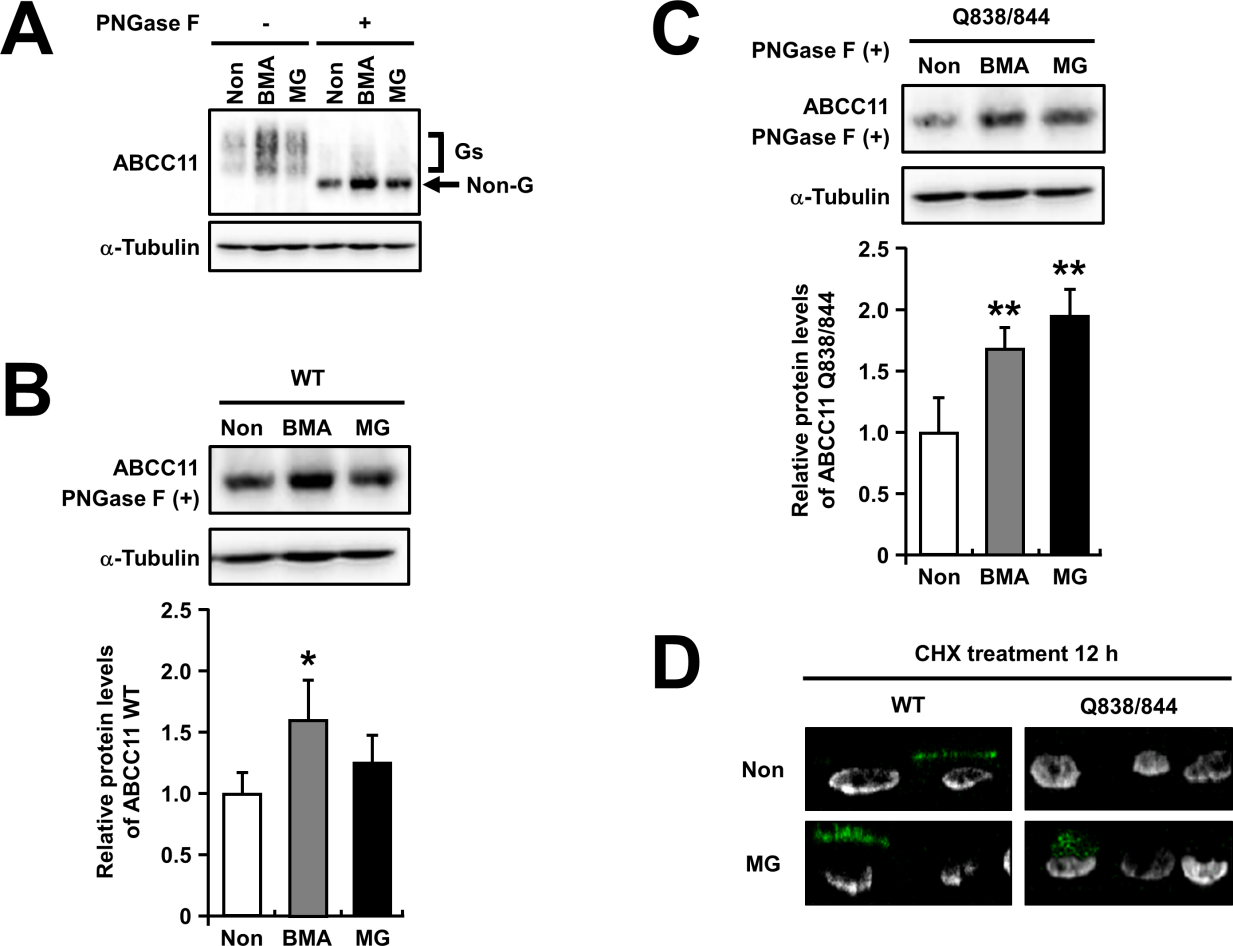
The effect of bafilomycin A_1_ or MG132 treatment on the protein levels of ABCC11. (A) Forty-eight hours after the transfection, MDCKII cells transiently expressing ABCC11 wild-type (WT) were cultured with 10 nM bafilomycin A_1_ (BMA) or 2 μM MG132 (MG) for a further 24 h. Then, cell lysates were subjected to immunoblotting after treatment with or without PNGase F. Gs, glycosylated forms; Non-G, non-glycosylated form. (B and C) Densitometoric analysis of protein levels of ABCC11 WT (B) and Q838/844 mutant (C) in BMA or MG-treated cells. After PNGase F treatment, cell lysates were subjected to immunoblotting. α-Tubulin: a loading control. The signal intensity ratio (ABCC11/α-tubulin) of the immunoreactive bands was determined and normalized to the non-treated control (Non). Data are expressed as mean ± S.D. *n* = 4. Statistical analyses for significant differences were performed according to Student’s *t* test (*, *P* < 0.05; **, *P* < 0.01 compared with control). (D) Z-sectioning images of cycloheximide (CHX)-treated MDCKII cells transiently expressing ABCC11 WT or Q838/844 mutant in the presence of MG132. Seventy-two hours after the transfection, MDCKII cells were cultured with 50 μM CHX in the presence or absence of 2 μM MG132 for further 12 h. Nuclei were stained with TO-PRO®-3 iodide (gray).

Next, we investigated the molecular basis related to the faster degradation of the Q838/844 mutant than ABCC11 WT. Since most of the membrane proteins are supposed to be degraded in either the lysosomal pathway or proteasomal pathway, we examined the effect of inhibitors for each proteolysis pathway on the ABCC11 protein levels. Forty-eight hours after the transfection, MDCKII cells expressing each ABCC11 protein were incubated for a further 24 h in the presence of 10 nM bafilomycin A_1_ (BMA) or 2 μM MG132, and then subjected to immnoblotting analysis (**Fig 6**). The protein level of ABCC11 WT was significantly enhanced, more than 1.5-fold, when the cells were treated with BMA, whereas there was no significant difference with MG132 treatment (**Fig 6A and 6B**), suggesting that ABCC11 WT was majorly degraded by the BMA-sensitive lysosomal pathway in MDCKII cells. On the other hand, in the case of the ABCC11 Q838/844 mutant, the protein level was increased about 2-fold when the cells were treated with not only BMA but also MG132 (**Fig 6C**). To examine the effect of MG132 treatment on the ABCC11 proteins that were mainly localized on the apical membrane of MDCKII cells (**Fig 4**), 72 h after the transfection of each ABCC11-expressing plasmid when the intracellular accumulation of each overexpressed ABCC11 protein was scarcely detected with confocal microscopic observations (**S4 Fig**), MDCKII cells were treated with 50 μM CHX in the presence or absence of 2 μM MG132 for further 12 h (**Fig 6D**). Z-sectioning images of the cells shows that most of ABCC11 WT was detectable as apical membrane protein after the CHX treatment and that the additional MG132 treatment did not cause the apparent change of the localization of ABCC11 WT protein. With the same condition of the confocal microscopic observation, ABCC11 Q838/844 was almost undetectable after the CHX treatment. On the other hand, when treated with both CHX and MG132, the intracellular signals of ABCC11 Q838/844 were detected. These results suggest that the MG132-sensitive proteasomal pathway was enhanced in the Q838/844 mutant, resulting in the lower protein levels (**Fig 3**) and higher degradation rate (**Fig 5**) of this *N*-glycan-deficient mutant as compared with WT.

### Low glucose decreases ABCC11 glycosylation and protein level

**Fig 7:**
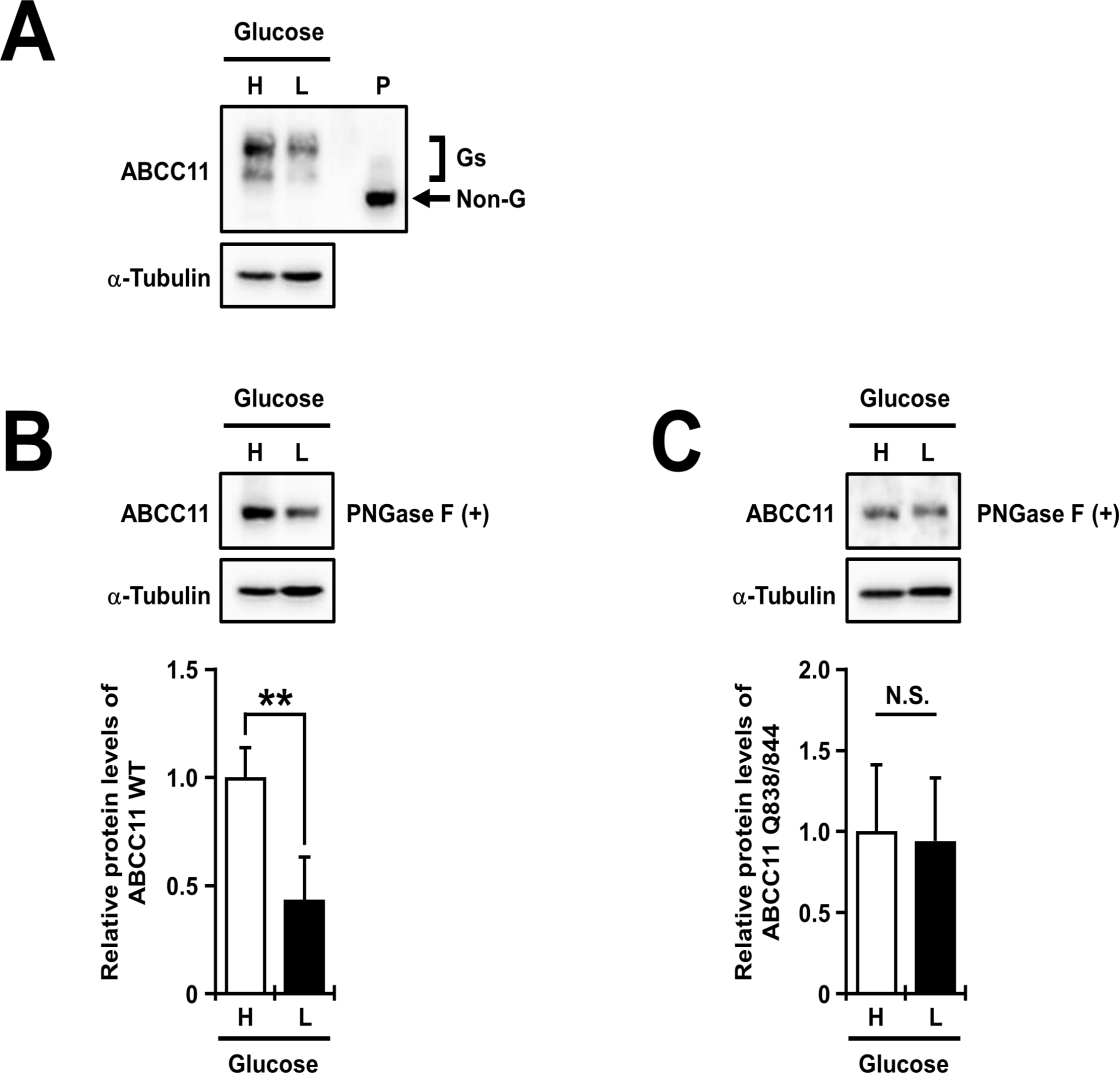
The effect of extracellular glucose on the protein levels of ABCC11. (A and B) The decrease of the glycosylation and the protein levels of ABCC11 wild-type (WT) expressed in MDCKII cells cultured with a low-glucose medium. (C) The little effect of low-glucose culture on the protein levels of the Q838/844 mutant expressed in MDCKII cells. Forty-eight hours after the transfection, mostly confluent MDCKII cells transiently expressing ABCC11 were cultured with high (H) or low (L) glucose medium for further 24 h. Then, cell lysates were prepared and subsequently subjected to immunoblotting after treatment without (A) or with (B, C) PNGase F. The immunoreactive bands disappeared with PNGase F treatment, corresponding to the glycosylated forms (Gs) of ABCC11 protein. α-Tubulin: a loading control. In densitometoric analyses of protein levels of ABCC11 WT and the Q838/844 mutant, the signal intensity ratio (ABCC11/α-tubulin) of the immunoreactive bands was determined and normalized to the control (high glucose) level. P: PNGase F-treated ABCC11 WT as a control for the band position of the non-glycosylated form of ABCC11 (details are described in *Materials and Methods*). Data are expressed as mean ± S.D. *n* = 4. Statistical analyses for significant differences were performed according to Student’s *t* test (**, *P* < 0.01). N.S.: not significantly different among groups.

Recent studies show that *N*-glycan biosynthesis depends on the utility of sugar substrates such as UDP-GlcNAc, an intracellular metabolite of glucose in the hexosamine pathway [40, 41]. This metabolic pathway is enhanced by an increase of glucose flux into cells [42]. In this context, we hypothesized that the change of extracellular glucose level would affect, as a glycoprotein, the protein levels of matured ABCC11. To verify this hypothesis, 48 h after the transfection, MDCKII cells were further incubated with high (22.5 mM) or low (5 mM) glucose medium for 24 h. Immunoblotting analysis revealed that the amount of glycosylated ABCC11 WT in MDCKII cells cultured in the low glucose condition was significantly lower than that in the high glucose condition (**Fig 7A**). The similar tendency in the total protein levels of ABCC11 WT was confirmed by PNGase F treatment (**Fig 7B**). On the other hand, there was no significant difference in the protein levels of the Q838/844 mutant in MDCKII cells between the different glucose conditions (**Fig 7C**). In addition, the protein levels of Q838/844 mutant were not higher than those of ABCC11 WT in each glucose condition (**S5 Fig**). Further immunoblotting analyses revealed that the amount of glycosylated gp135, an endogenous apical membrane protein, in MDCKII cells cultured in the low glucose condition was significantly lower than that in the high glucose condition (**S5 Fig**). In the case of ABCG2 and GLUT-9b transiently expressed in MDCKII cells, there was no difference in their protein levels between the different glucose conditions (**S5 Fig**). Considering that mRNA expression of each ABCC11 from vectors was exogenously regulated by the CMV promoter in these experiments, the effect of glucose on the ABCC11 protein levels would not be due to transcriptional regulation. Hence, our data suggest that the decrease of extracellular glucose would suppress the glycosylation process of ABCC11, resulting in the decrease in the protein levels of ABCC11 WT.

## Discussion

In the current study, we investigated the role of *N*-linked glycosylation in the regulation of ABCC11 protein expressed in polarized MDCKII cells. Biochemical analyses indicated that the disruption of *N*-linked glycosylation decreased the protein stability of ABCC11 (**Figs 2 and 3, S2 Fig**). Actually, the *N*-linked glycan-deficient Q838/844 mutant was degraded faster than WT (**Fig 5**). On the other hand, the absence of *N*-linked glycan did not affect the apical localization of ABCC11 in MDCKII cells (**Fig 4**). Although *N*-linked glycosylation has been shown to play an important role in the apical localization of a number of proteins, some exceptional cases have also been reported. For example, ABCG2, a physiologically important ABC transporter [43–45], does not require *N*-linked glycosylation for its apical localization in polarized LLC-PK1 cells [31] and MDCKII cells (**S3 Fig**), whereas its *N*-linked glycan contributes to the stabilization of ABCG2 protein [27], which is confirmed again in the present study (**S3 Fig**). The similar result is also the case with apical sodium-dependent bile acid transporter (ASBT) [25]. These observations support our results demonstrating that *N*-linked glycan would increase the protein stability of ABCC11, while it would not be essential for apical trafficking of ABCC11. Multiple alignment analysis revealed that the *N*-linked glycosylation sites corresponding to Asn838 and Asn844 in human ABCC11 are highly conserved in various species (**Table 1**). The presence and combination of these two glycans might be important for the stability and/or function of ABCC11 expressed in human body.

**Table 1:** Partial amino acid sequences near the *N*-linked glycosylation sites in human ABCC11 and the corresponding sequences of its orthologues in mammals.

Species	Homology^1^	Sequences	Positions	Total amino acids	Access No.
Human	100	WLEQGSGT**N**SSRES**N**GTMADL	830–850	1382	NP_115972
Chimpanzee	98	WLEQGSGT**N**SSRES**N**GTMADL	830–850	1382	XP_009429028
Gorilla	98	WLEQGSGT**N**SSRES**N**GTMADL	830–850	1382	XP_004057661
Monkey	91	WLEQGSGT**N**SSRES**N**GTTADP	830–850	1382	XP_011757555
Goat	72	WLGQGSGK**N**SSQES**N**WTTADP	827–847	1379	XP_005692011
Cattle	70	WLGQGSGN**N**SSQES**N**WTTADP	827–847	1255	XP_010813025
Horse	73	WLEQGSGA**N**SSQES**N**RTPADP	833–853	1409	XP_003364699
Dog	75	WLEQGSGT**N**SSQEN**N**GTTADP	832–852	1384	XP_003638982
Cat	75	WLQQGSGT**N**SSQES**N**GTTADP	833–853	1385	XP_003998059
Rabbit	73	WLQQGSGT**N**SSRGS**N**SSSADP	831–851	1387	XP_002721399
Rat	-	-	-	-	None^2^
Mouse	-	-	-	-	None^2^

^1^ Homology (%) to the full length of human ABCC11 protein.

^2^ No orthologous gene has been reported to date. Each sequence was obtained from NCBI Protein Database. Reference sequence ID was described in the column of Access No. Putative sites of *N*-linked glycosylation (Asn-X-Thr/Ser, X ≠ Pro) are indicated by underlining.

The results shown in this study lead our deeper understanding of the role of *N*-linked glycosylation in the ABCC11 regulation. In our previous study using non-polarized Flp-In-293 cells which were generated from HEK293 cells [46], we could determine the *N*-linked glycosylation sites in ABCC11 protein [16]. At that time, the effect of *N*-linked glycan on the protein level of ABCC11 was not as clearly observed as compared with the current study, which was probably due to less trafficking of ABCC11 protein to plasma membrane (PM) in non-polarized Flp-In-293 cells than in polarized MDCKII cells. Actually, in the present study, little signal indicating the localization of ABCC11 WT on the PM of non-polarized HEK293 cells was detected (**S6 Fig**), whereas ABCC11 WT derived from the same plasmid construct was mainly localized on the PM of MDCKII cells (**Fig 4**). The similar apical localization of ABCC11 was reported in the previous study using the MDCKII cells stably transfected with non-tagged ABCC11 [17], suggesting the presence of EGFP-tag would rarely affect the localization of ABCC11 protein. ABCC11 Q838/844 mutant was also mainly localized on the PM in MDCKII cells. Considering the higher clearance rate of the Q838/844 mutant than that of WT (**Figs 5 and 6D**), the *N*-linked glycan-deficient mutant seems to be eliminated from the cell surface rapidly as compared with ABCC11 WT. This interpretation is in line with the similar results observed in several PM proteins including cystic fibrosis transmembrane conductance regulator (CFTR), also known as ABCC7, which belongs to the same (ABCC) subfamily of ABC transporter super-family that includes ABCC11 [25, 27, 47]. Hence, *N*-linked glycan would enhance the protein level of ABCC11 localized on PM.

We found that *N*-linked glycosylation increases ABCC11 protein stability and glucose condition may have an impact on the process. In this regard, our data show that the protein levels of *N*-glycosylated ABCC11 WT were sensitive to the change of glucose condition, whereas those of glycosylation-deficient ABCC11 Q838/844 were not sensitive (**Fig 7**). In addition, there were two types of glycoproteins, sensitive and non-sensitive to glucose conditions (**S5 Fig**). Although the current study did not reveal the molecular basis that determines the sensitivity of glycoprotein to glucose conditions, our data suggest that high levels of glucose would enhance ABCC11 glycosylation leading to an increase in ABCC11 protein levels, likely via promoting its protein stability. The similar results were obtained in the following studies both *in vitro* and *in vivo*: (i) *in vitro* exposure to glucose increased the mature glycosylated ASBT protein, but not the core glycosylated ASBT, probably via promoting its stability [25]; (ii) in diabetes model rat, mature glycosylation form of native UT-A1, an urea transporter showing apical localization in polarized cells, was increased with the change in its glycosylation profile [48].

The present study has a limitation that our experimental approach was restricted to the overexpression of ABCC11 due to the non-availability of appropriate cell lines that have the endogenous expression of ABCC11. Considering that the removal of glycosylation site could trigger an ERAD-mediated proteolysis, and that the overexpressed proteins from transfected DNA could evade ERAD sometimes, constitutionally glycosylation-deficient ABCC11 might not be expressed on PM in physiological condition as we observed in the present study. On the other hand, even the endogenous PM protein that is recognized as an ERAD substrate such as ΔF508 CFTR/ABCC7 is reportedly localized on the PM in the ΔF508 homozygous humans [49, 50]. Thus, we have realized that the PM localization of ABCC11 Q838/844 mutant did not mean that this mutant was not an ERAD substrate. In other words, we could not determine whether or not the Q838/844 mutant was degraded by ERAD.

We report here that the *N*-linked glycan would not be prerequisite for the apical localization of ABCC11, at least, in MDCKII cells. However, the molecular mechanisms regulating the apical targeting of ABCC11 remain to be addressed. Although little information is available, in the case of some other ABCC subfamily proteins such as ABCC2/MRP2, ABCC4/MRP4, and ABCC7/CFTR, an interaction between these ABC transporters and PSD95/Dlg/ZO-1 (PDZ) adapter proteins is supposed to be important for their sorting and/or stabilization on the apical membrane, at least *in vitro* [51–54]. Actually, those ABCC proteins have a class I PDZ-binding motif (-S/T-X-Ф-COOH; X denotes any amino acid, Ф denotes a hydrophobic acid such as Val, Ile and Leu) at their C-terminus—a consensus sequence in most transporters expressed on the apical membranes [51]. Although ABCC11 does not have the consensus sequence, its C-terminal sequence (-S-S-L-R-COOH) is highly similar to the PDZ motif. Thus, this PDZ-like sequence might be important for the intracellular regulation of ABCC11 via the interaction with PDZ proteins. Since ABCC11 is a protein of medical importance, any regulatory mechanism controlling the localization of ABCC11 would be the next target of future investigation.

Besides the elucidation of the role of *N*-linked glycosylation in ABCC11 protein stability, the current study has one clinical implication. Namely, chronic hyperglycemia might exacerbate axillary osmidrosis via the increase of matured ABCC11, since the current study implies that high levels of glucose would enhance the glycosylation of ABCC11, resulting in the increase in ABCC11 protein levels (**Fig 7**). Indeed, ABCC11 is responsible for the axillary osmidrosis risk probably via the development of human apocrine glands in axilla [55]. Considering the results that the decrease of glucose in culture medium decreased the protein level of ABCC11 (**Fig 7A and 7B**), maintaining a normal blood glucose level as low as practical might be an effective way to prevent and/or attenuate axillary osmidrosis. Unfortunately, the lack of an *Abcc11* gene in rodents gives us a limitation for *in vivo* investigation in the current study (**Table 1**), and there is no epidemiological study about the relationship between the axillary osmidrosis phenotype and blood glucose level to our knowledge. Further investigations using ABCC11-transgenic mice and clinical samples would shed light on the implications.

## Conclusions

In the current study, we characterized the expression and apical localization of ABCC11 protein with *N*-linked glycan in polarized MDCKII cells. Our results demonstrate that *N*-linked glycosylation is important for the protein stability of ABCC11, whereas it is not essential for the apical localization of ABCC11. These results suggest that the inhibition and/or disruption of *N*-glycan attached to ABCC11 would be a new approach for the treatment of axillary osmidrosis. In addition, physiological alteration of the glucose condition in the human body might affect the axillary osmidrosis phenotype. Further research to verify this potential association between glucose and the ABCC11-related phenotypes would be important.

## Supporting Information

S1 Fig Immunoblotting analysis of non-tagged ABCC11 and ABCC11-EGFP transiently expressed in HEK293 cells and MDCKII cells. HEK293 cells and MDCKII cells were transiently transfected with non-tagged ABCC11 wild-type (WT) or ABCC11 WT-EGFP. Cell lysates were prepared 72 h after the transfection, and subsequently subjected to immunoblotting using the anti-ABCC11 antibody or the anti-EGFP antibody after treatment with or without PNGase F. Each exposure time for signal detection is indicated in parentheses. Mock: non-inserted pcDNA3.1/hygro(-) plasmid, EGFP: non-inserted pEGFP-N1 plasmid, α-Tubulin: a loading control. Anti-ABCC11 antibody could detect both non-tagged ABCC11 and ABCC11-EGFP expressed in HEK293 cells. On the other hand, these proteins expressed in MDCKII were undetectable with the anti-ABCC11 antibody, whereas ABCC11-EGFP expressed in MDCKII was detected with anti-EGFP antibody. Relationship between the band intensity and exposure time in each immunoblotting image suggest that the anti-EGFP antibody was more sensitive than the anti-ABCC11 antibody, and that the total protein levels of transfected product in MDCKII cells were significantly lower than those in HEK293 cells.
(TIF)

S2 Fig The effect of tunicamycin, an *N*-linked glycosylation inhibitor, on lowering the protein levels of non-tagged ABCC11. (A) Effect of adenovirus infection on the viability of MDCKII cells. MDCKII cells were seeded at a density of 1.4 × 10^5^ cells/cm^2^ onto 12-well cell culture plate. Twelve hours after the seeding, the cells were infected with adenovirus at indicated MOIs. After 48 h of a further incubation, WST-8 assay was performed to evaluate the cellular viability in each well. Data are expressed as mean ± S.D. *n* = 6. Statistical analyses for significant differences were performed according to Bartlett’s test, followed by Dunnett’s test. N.S.: not significantly different as compared with non-infected (0 MOI) control. (B) Immunoblotting analysis of non-tagged ABCC11 expressed in adenovirus-infected MDCKII cells. Forty-eight hours after the adenovirus infection at non-toxic 200 MOI, the MDCKII cells transiently expressing non-tagged ABCC11 WT were cultured with fresh medium with or without tunicamycin (TNM) (4.0 μg/mL) for further 24 h. Subsequently, cell lysate samples were prepared and treated with or without PNGase F, and then subjected to immunoblotting using the anti-ABCC11 antibody. The protein levels of non-tagged ABCC11 in the tunicamycin-treated cells were significantly lower (15% ± 14% as compared with non-treated control, *n* = 3) than those in non-treated cells. α-Tubulin: a loading control. Results are representative of three independent sample sets.
(TIF)

S3 Fig The effect of the disruption of *N*-linked glycosylation sites on the protein levels and localization of ABCG2. (A) Immunoblotting and densitometoric analysis of protein levels of ABCG2 WT and glycosylation-deficient Q596 mutants expressed in MDCKII cells 72 h after the transfection. Cell lysate samples were prepared and treated with or without PNGase F, and then subjected to immunoblotting. The immunoreactive bands, corresponding to the glycosylated forms (Gs, glycosylation at N596) of the ABCG2 protein, disappeared with PNGase F treatment. The signal intensity ratio (ABCG2/α-tubulin, a loading control) of the immunoreactive bands corresponding to non-glycosylated (Non-G) ABCG2 was determined and normalized to the WT level. Data are expressed as mean ± S.D. *n* = 3. Statistical analyses for significant differences were performed according to Student’s *t* test (*, *P* < 0.05). (B) Apical localization of ABCG2 and Q596 mutant expressed in MDCKII cells 72 h after the transfection. An endogenous apical membrane marker gp135 was immunostained using the anti-gp135 antibody (red). Nuclei were stained with TO-PRO®-3 iodide (gray). All panels show the Z-sectioning images. As expected, each ABCG2 protein was localized on the apical membrane of MDCKII cells, which is consistent with previous reports as described in the main text.
(TIF)

S4 Fig Confocal microscopic observation of ABCC11 WT and Q838/844 mutant expressed in MDCKII cells 72 h after the plasmid transfection. Overexpressed ABCC11 WT and Q838/844 mutant were localized on the apical membrane of MDCKII cells. On the other hand, there is little signal indicating the intracellular accumulation of each protein. Nuclei were stained with TO-PRO®-3 iodide (gray). The upper and middle panels are en face images focused at the top and middle of the cells, respectively. The bottom panels show the Z-sectioning images (XZ). Bars: 5 μm.
(TIF)

S5 Fig The effect of extracellular glucose on the protein levels of apical membrane proteins. (A) ABCC11 wild-type (WT) and N838/844 mutant, (B) endogenous gp135, (C) ABCG2 WT, and (D) GLUT-9b WT. Forty-eight hours after the transfection, mostly confluent MDCKII cells were cultured with high (H) or low (L) glucose medium for further 24 h. Then, cell lysates were prepared and subsequently subjected to immunoblotting after treatment with or without PNGase F. The immunoreactive bands disappeared with PNGase F treatment, corresponding to the glycosylated form(s) (G(s)) of each protein. α-Tubulin: a loading control. (B-D) In densitometoric analyses of protein levels of each apical membrane protein, the signal intensity ratio (target protein/α-tubulin) of the immunoreactive bands corresponding to the non-glycosylated (non-G) form was determined and normalized to the control (high glucose) level. Data are expressed as mean ± S.D. *n* = 4 (B), 3 (C and D). Statistical analyses for significant differences were performed according to Student’s *t* test (*, P < 0.05). N.S.: not significantly different among groups.
(TIF)

S6 Fig Localization of ABCC11 in non-polarized HEK293 cells. HEK293 cells were transiently transfected with ABCC11 wild-type (WT)-EGFP, and imaged by confocal microscopy 72 h after the transfection. In order to visualize the plasma membranes (PMs), cells were pre-treated with CellMask™ Orange Plasma Membrane Stain (Life technologies) (1:2000 diluted in PBS (-)) for 10 min at room temperature. Bars: 5 μm.
(TIF)
