# Supplementary figures and images for: Regulation of the Axillary Osmidrosis-Associated ABCC11 Protein Stability by N-Linked Glycosylation: Effect of Glucose Condition
Source: PLoS One. 2016 Jun 9;11(6):e0157172. doi: 10.1371/journal.pone.0157172 (PMC4900533; doi:10.1371/journal.pone.0157172)

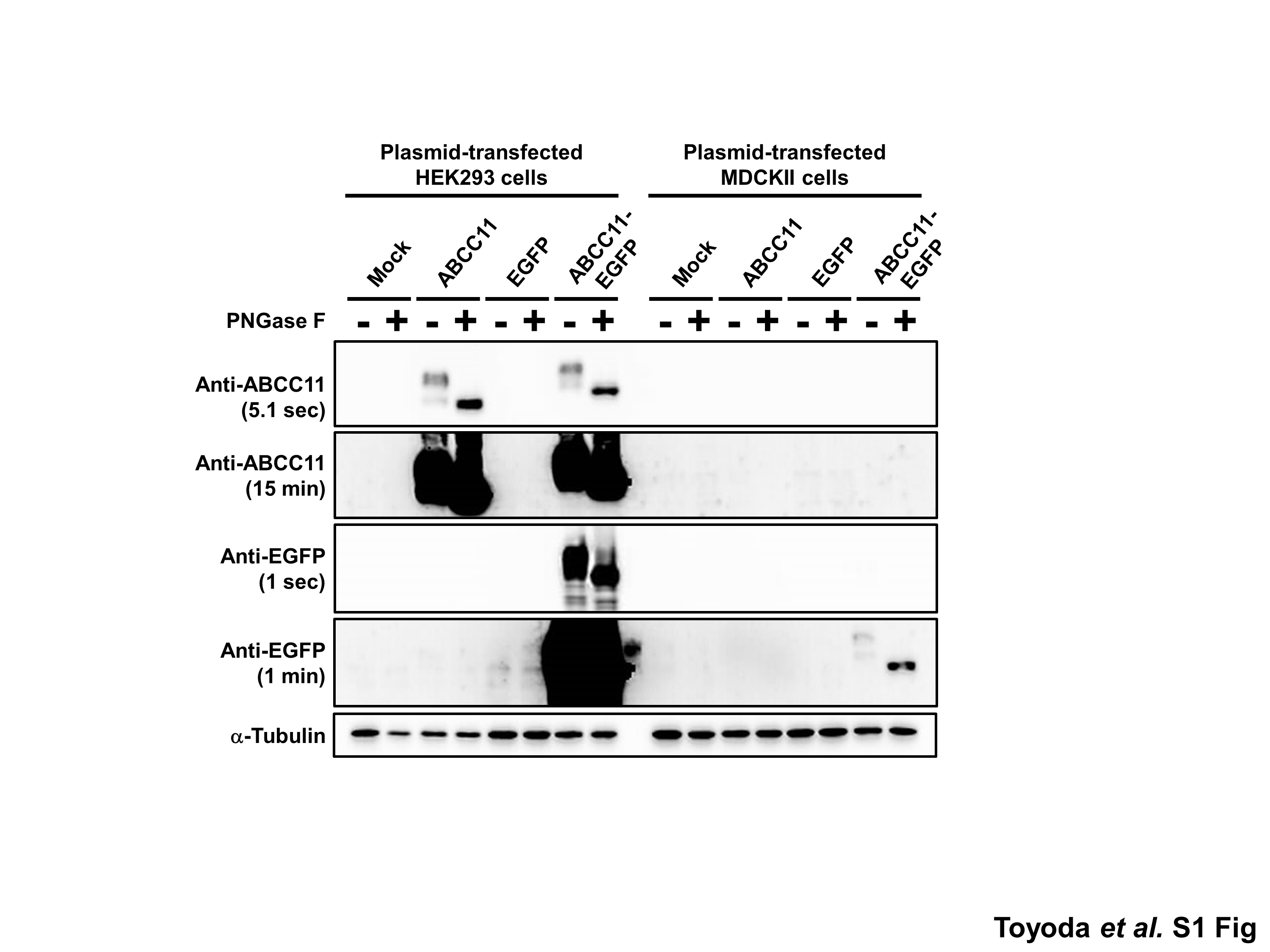

Supplement: S1 Fig — HEK293 cells and MDCKII cells were transiently transfected with non-tagged ABCC11 wild-type (WT) or ABCC11 WT-EGFP. Cell lysates were prepared 72 h after the transfection, and subsequently subjected to immunoblotting using the anti-ABCC11 antibody or the anti-EGFP antibody after treatment with or without PNGase F. Each exposure time for signal detection is indicated in parentheses. Mock: non-inserted pcDNA3.1/hygro(-) plasmid, EGFP: non-inserted pEGFP-N1 plasmid, α-Tubulin: a loading control. Anti-ABCC11 antibody could detect both non-tagged ABCC11 and ABCC11-EGFP expressed in HEK293 cells. On the other hand, these proteins expressed in MDCKII were undetectable with the anti-ABCC11 antibody, whereas ABCC11-EGFP expressed in MDCKII was detected with anti-EGFP antibody. Relationship between the band intensity and exposure time in each immunoblotting image suggest that the anti-EGFP antibody was more sensitive than the anti-ABCC11 antibody, and that the total protein levels of transfected product in MDCKII cells were significantly lower than those in HEK293 cells. (TIF) [file pone.0157172.s001.TIF]

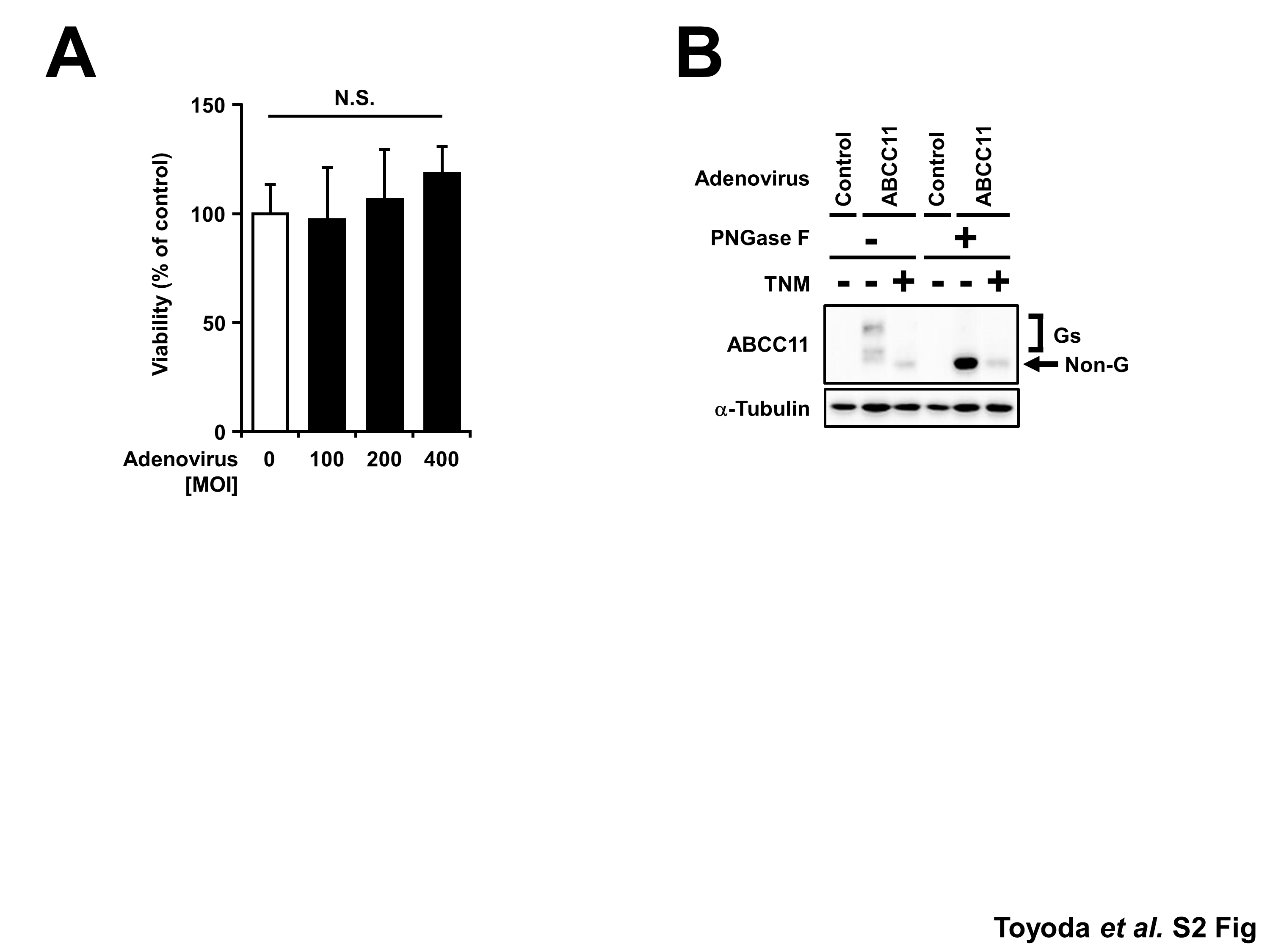

Supplement: S2 Fig — (A) Effect of adenovirus infection on the viability of MDCKII cells. MDCKII cells were seeded at a density of 1.4 × 10^5^ cells/cm2 onto 12-well cell culture plate. Twelve hours after the seeding, the cells were infected with adenovirus at indicated MOIs. After 48 h of a further incubation, WST-8 assay was performed to evaluate the cellular viability in each well. Data are expressed as mean ± S.D. n = 6. Statistical analyses for significant differences were performed according to Bartlett’s test, followed by Dunnett’s test. N.S.: not significantly different as compared with non-infected (0 MOI) control. (B) Immunoblotting analysis of non-tagged ABCC11 expressed in adenovirus-infected MDCKII cells. Forty-eight hours after the adenovirus infection at non-toxic 200 MOI, the MDCKII cells transiently expressing non-tagged ABCC11 WT were cultured with fresh medium with or without tunicamycin (TNM) (4.0 μg/mL) for further 24 h. Subsequently, cell lysate samples were prepared and treated with or without PNGase F, and then subjected to immunoblotting using the anti-ABCC11 antibody. The protein levels of non-tagged ABCC11 in the tunicamycin-treated cells were significantly lower (15% ± 14% as compared with non-treated control, n = 3) than those in non-treated cells. α-Tubulin: a loading control. Results are representative of three independent sample sets. (TIF) [file pone.0157172.s002.TIF]

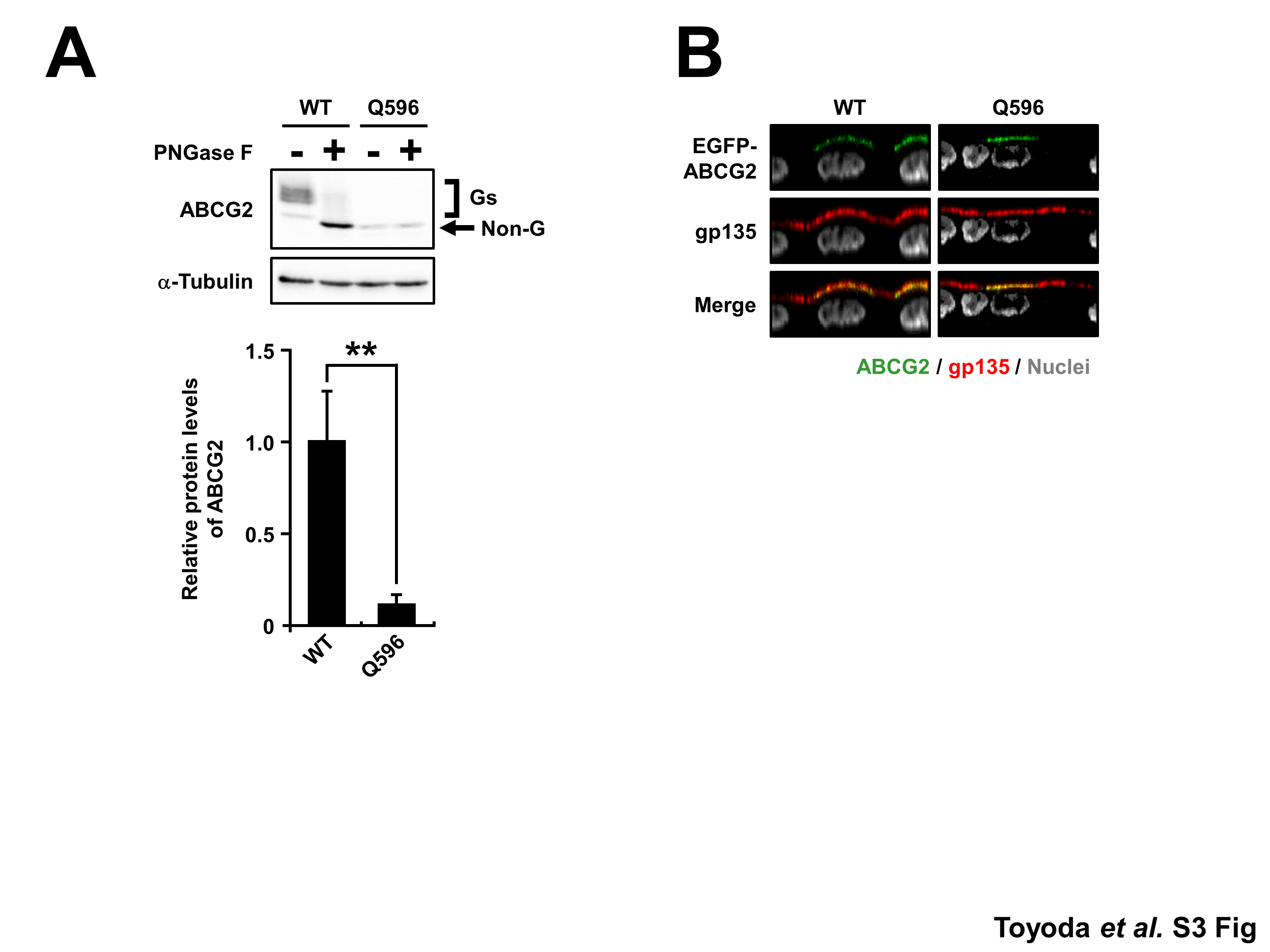

Supplement: S3 Fig — (A) Immunoblotting and densitometoric analysis of protein levels of ABCG2 WT and glycosylation-deficient Q596 mutants expressed in MDCKII cells 72 h after the transfection. Cell lysate samples were prepared and treated with or without PNGase F, and then subjected to immunoblotting. The immunoreactive bands, corresponding to the glycosylated forms (Gs, glycosylation at N596) of the ABCG2 protein, disappeared with PNGase F treatment. The signal intensity ratio (ABCG2/α-tubulin, a loading control) of the immunoreactive bands corresponding to non-glycosylated (Non-G) ABCG2 was determined and normalized to the WT level. Data are expressed as mean ± S.D. n = 3. Statistical analyses for significant differences were performed according to Student’s t test (*, P < 0.05). (B) Apical localization of ABCG2 and Q596 mutant expressed in MDCKII cells 72 h after the transfection. An endogenous apical membrane marker gp135 was immunostained using the anti-gp135 antibody (red). Nuclei were stained with TO-PRO®-3 iodide (gray). All panels show the Z-sectioning images. As expected, each ABCG2 protein was localized on the apical membrane of MDCKII cells, which is consistent with previous reports as described in the main text. (TIF) [file pone.0157172.s003.TIF]

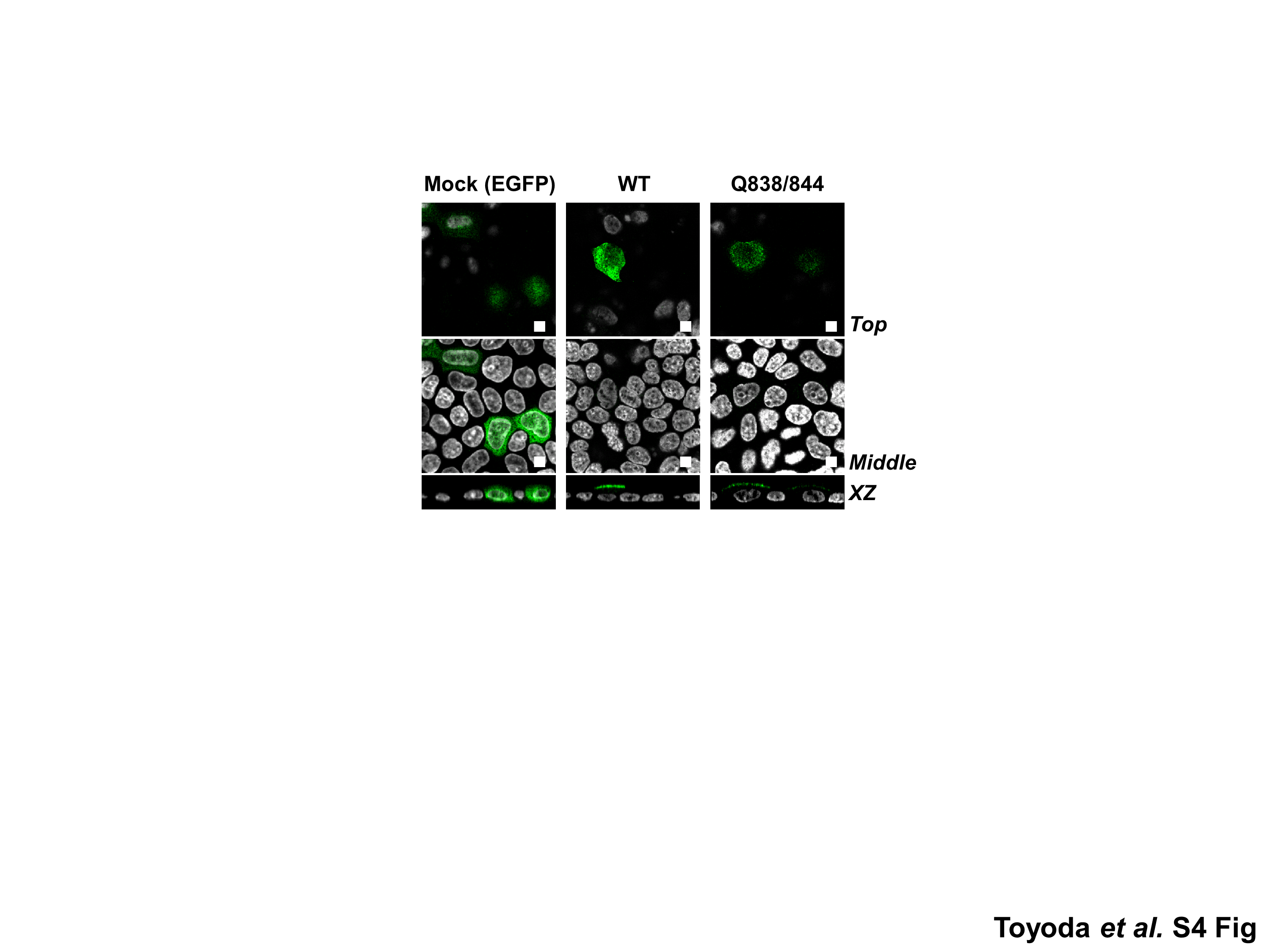

Supplement: S4 Fig — Overexpressed ABCC11 WT and Q838/844 mutant were localized on the apical membrane of MDCKII cells. On the other hand, there is little signal indicating the intracellular accumulation of each protein. Nuclei were stained with TO-PRO®-3 iodide (gray). The upper and middle panels are en face images focused at the top and middle of the cells, respectively. The bottom panels show the Z-sectioning images (XZ). Bars: 5 μm. (TIF) [file pone.0157172.s004.TIF]

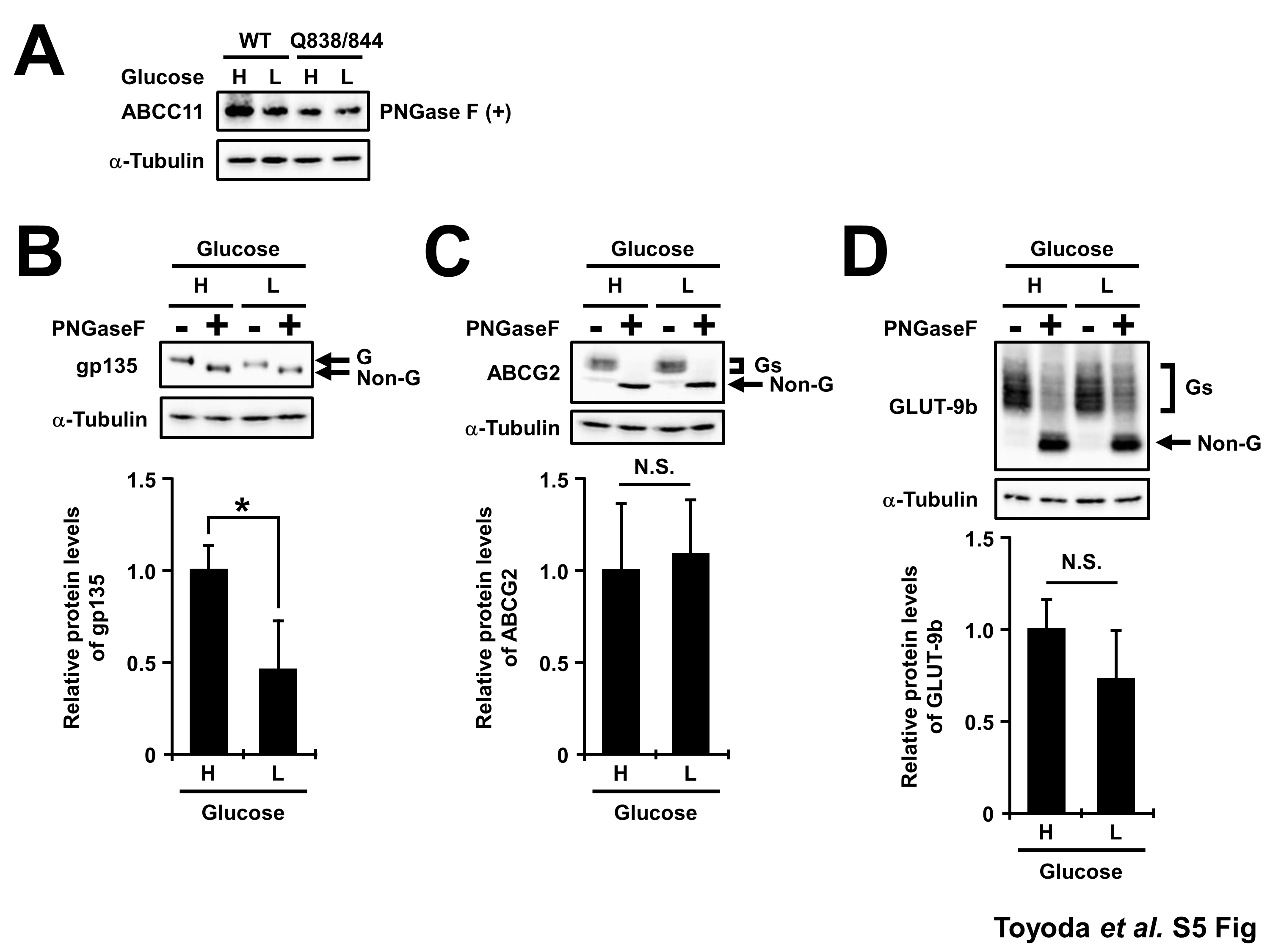

Supplement: S5 Fig — (A) ABCC11 wild-type (WT) and N838/844 mutant, (B) endogenous gp135, (C) ABCG2 WT, and (D) GLUT-9b WT. Forty-eight hours after the transfection, mostly confluent MDCKII cells were cultured with high (H) or low (L) glucose medium for further 24 h. Then, cell lysates were prepared and subsequently subjected to immunoblotting after treatment with or without PNGase F. The immunoreactive bands disappeared with PNGase F treatment, corresponding to the glycosylated form(s) (G(s)) of each protein. α-Tubulin: a loading control. (B-D) In densitometoric analyses of protein levels of each apical membrane protein, the signal intensity ratio (target protein/α-tubulin) of the immunoreactive bands corresponding to the non-glycosylated (non-G) form was determined and normalized to the control (high glucose) level. Data are expressed as mean ± S.D. n = 4 (B), 3 (C and D). Statistical analyses for significant differences were performed according to Student’s t test (*, P < 0.05). N.S.: not significantly different among groups. (TIF) [file pone.0157172.s005.TIF]

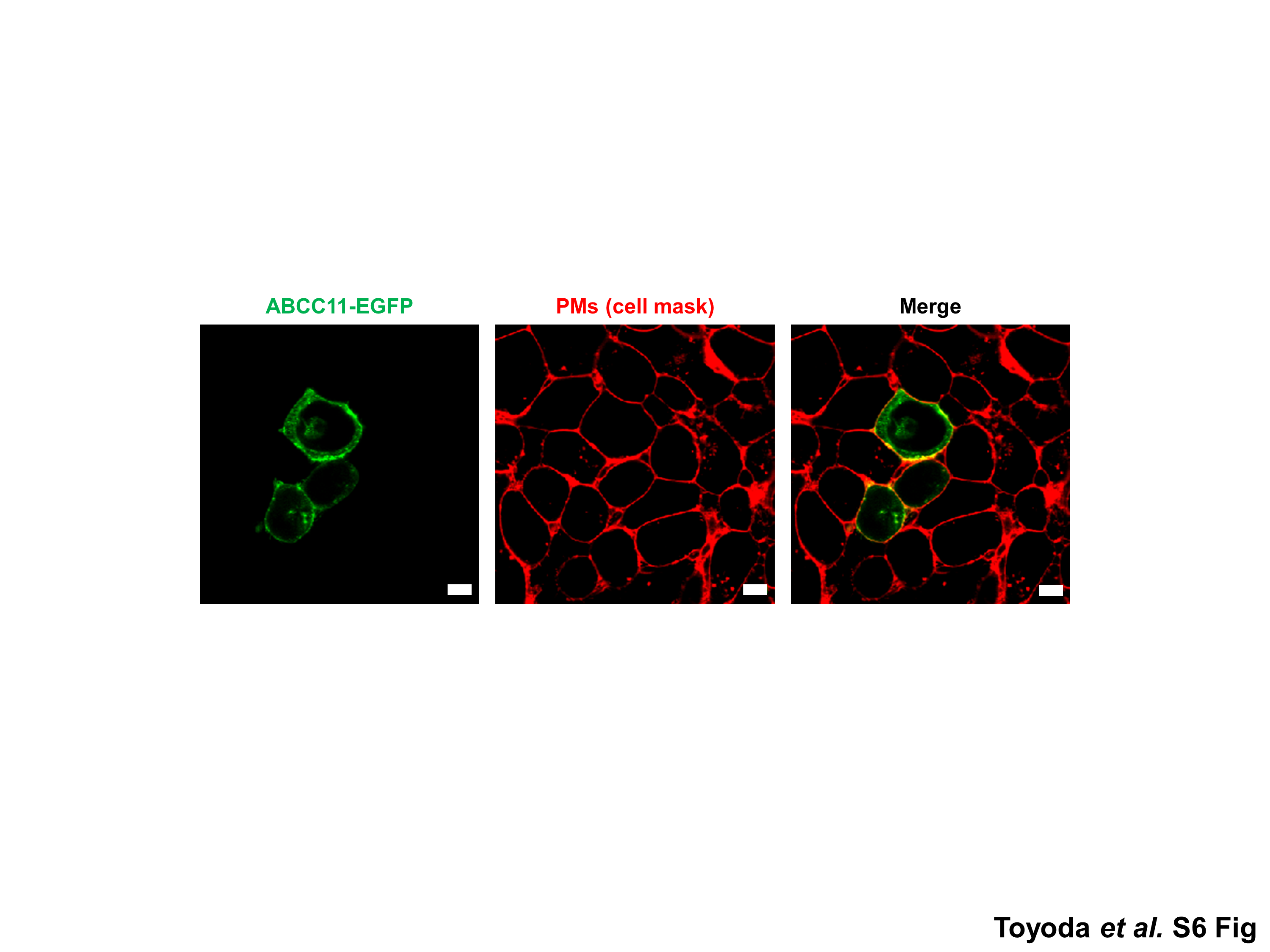

Supplement: S6 Fig — HEK293 cells were transiently transfected with ABCC11 wild-type (WT)-EGFP, and imaged by confocal microscopy 72 h after the transfection. In order to visualize the plasma membranes (PMs), cells were pre-treated with CellMask™ Orange Plasma Membrane Stain (Life technologies) (1:2000 diluted in PBS (-)) for 10 min at room temperature. Bars: 5 μm. (TIF) [file pone.0157172.s006.TIF]
